# Effects of DNA-targeted ionizing radiation produced by 5-[^125^I]iodo-2'-deoxyuridine on global gene expression in primary human cells

**DOI:** 10.1186/1471-2164-8-192

**Published:** 2007-06-26

**Authors:** Mykyta V Sokolov, Ronald D Neumann, Igor G Panyutin

**Affiliations:** 1 Nuclear Medicine Department, Clinical Center, National Institutes of Health, 9000 Rockville Pike, Bethesda, 20892, Maryland, USA

## Abstract

**Background:**

This study assesses the whole-genome gene expression changes in a panel of primary human cell lines in response to DNA damage mediated by decay of DNA-incorporated radioiodinated thymidine analog 5-[^125^I]iodo-2'-deoxyuridine (^125^I-IUdR). Three normal human cell lines of different origin, namely, gingival fibroblasts AG09319, fetal skin fibroblasts GM05388 and neonatal foreskin epidermal keratinocytes (NHFK) were used in this study. DNA molecules were radiolabeled by incubation of cells in culture in a medium supplemented with either 3.7 kBq/ml or 18.5 kBq/ml of ^125^I-IUdR for 24 h followed by incubation in IUdR-free medium for additional 24 hours. Each experiment was carried out in quadruplicate. ^125^I-IUdR uptake was monitored by measuring DNA-associated radioactivity. The whole-genome gene expression changes were evaluated using Agilent Human Whole Genome oligo microarrays containing 44,290 elements representing all known and predicted human genes. DNA microarray dataset was independently partially validated with quantitative real-time PCR (RT-PCR).

**Results:**

AG09319 gingival cells in culture responded to ^125^I-IUdR treatment by changing the expression level of 335 genes in total, whereas under the same conditions GM05388 and NHFK cells differentially expressed 49 genes and 27 genes, respectively. However, for GM05388 cells the number of differentially expressed genes increases with the rise of ^125^I-IUdR concentrations in cell culture media. The key up-regulated biological processes in a chosen panel of cell lines concern the regulation of protein kinase activities and/or cell death. Genes repressed in response to ^125^I-IUdR treatment are involved in cytokinesis, M phase of the cell cycle, chromosome architecture and organization, DNA metabolism, DNA packaging, DNA repair and response to DNA damage. Despite the disparate nature of the gene patterns elicited by ^125^I-induced DNA damage among the different cell lines, the differentially expressed transcripts reveal strikingly non-random chromosomal distribution in all the cell lines we used.

**Conclusion:**

Our data suggest that DNA-targeted ionizing radiation produced by ^125^I-IUdR results in changes in expression of only a limited subset of genes in primary human cells. The responsive genes are distributed non-randomly among the chromosomes; and a significant fraction of them is p53-dependent in the transcriptional regulation.

## Background

Exposure to ionizing radiation (IR) is inevitable occurrence for human beings. A variety of sources of IR, among them environmental, accidental and medical being the most important, contribute to a collective absorbed dose of exposure to IR posing a challenge to human health and well-being. The deleterious effects of IR are primarily due to its known carcinogenic, mutagenic and cell-killing ability. One of the mechanisms underlying these effects is thought to involve changes in gene expression following IR exposure [1]. Recent technological advances and the completion of Human Genome Project enabled scientists to study global expression changes for tens of thousands of genes at a time using powerful DNA microarray assays. Many reports dealing with the identification of gene expression profiles in human cells following IR exposures were published so far, focusing on either high or low dose-rate X-ray or gamma-ray exposures, responses in tumor or normal cells; or, alternatively, comparing effects of IR with other genotoxic agents [2-4]. However, very little is known about whole-genome transcriptional response of human cells to internally positioned radionuclides; the use of which for diagnostic and therapeutic purposes constitutes the core of nuclear medicine practice. In addition, such knowledge would be vital to implement strategies aiming to minimize consequences of possible nuclear power plant accidents or "dirty bomb" radiologic terrorist attack in the future.

Auger electron emitters such as ^125^I have been proposed for use in cancer therapy quite a long time ago [5]. DNA-incorporated ^125^I-IUdR was shown to be very efficacious (5- to 7-log cell killing) in the radiotherapy of small-animal malignancies [6]. Hence, there is steady interest in developing the optimized protocols of ^125^I-IUdR administration and other Auger electron emitters for possible clinical trials in the future. However, a lack of comprehensive information on how this radionuclide may affect the biological processes in treated cells at the molecular level hampers progress in this application. In our previous work, we showed that labeling of normal IMR-90 human fibroblasts with ^125^I-IUdR triggers changes in gene expression of about ten times fewer genes as gamma-radiation exposures delivered in bioequivalent doses [7]. We suggested that the effect of IR on the changes in global gene expression depends in part on the distribution of energy depositions within the cell; and that DNA double-strand breaks (DSBs) may not be the only factor modulating changes in gene expression following irradiation.

Herein, we extend our studies of ^125^I-IUdR-induced changes in gene expression on three primary human cell lines. Our goal was to determine whether incorporation of ^125^I into DNA molecules of human cells is associated with specific transcriptional changes that globally can be used as a Auger effect "fingerprint," to identify functions of genes whose expressions are altered following ^125^I decay; to learn whether primary cells undergoing ^125^I-triggered DNA damage show similar or disparate transcriptional changes. By using oligo DNA microarrays to concurrently compare the expression of about 41,000 transcripts in radioactively labeled cells, we identified distinct transcriptional perturbations that characterize the cellular response to ^125^I-triggered DNA damage in human fibroblasts and NHFK. Remarkably, we found that while genes showing altered expression during^ 125^I postlabeling differ dramatically in these two types of cells, these genes were non-randomly distributed among different chromosomes in both types of cells.

## Results

### ^125^I-IUdR-mediated DNA double-strand break induction

The rate of incorporation of ^125^I-IUdR into cellular DNA was evaluated according to published protocols [8]. Decay of ^125^I results in the emission of an average of 21 short-range electrons most of them having energies below 1 keV [9]. Therefore, nearly all energy of IR released upon decays deposits within the few nanometers of the decay site [10]. With DNA-incorporated ^125^I, as in our study, this means that DNA molecules are the targets of IR-mediated damage whereas the energy deposition in the rest of the cell is less than 5 % of that in the nucleus [9]. Assuming that one decay of DNA-incorporated ^125^I produces one DSB, we estimated that AG09319 cells in culture accumulated about 59 DNA DSBs over the course of radiolabeling, GM05388 cell cultures accumulated 32 DNA DSBs in total, whereas NHFK cultures – about 30 DNA DSBs on average, per our radiolabeling protocol [11]. Hence, DNA molecules in targeted cell populations were irradiated at a rate inflicting approximately 1–2 DNA DSBs per hour.

### Gene expression changes mediated by DNA-incorporated ^125^I-IUdR

**Figure 1:**
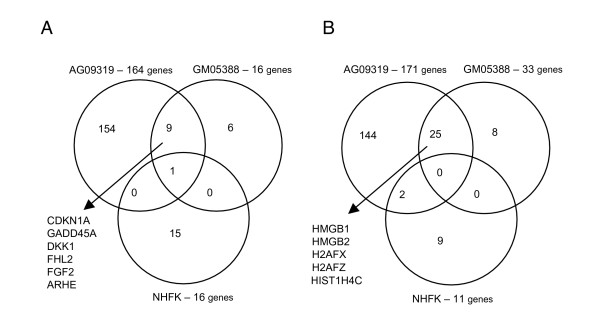
Venn diagram of ^125^I-IUdR-responsive sets of either induced (A) or repressed (B) genes.

DNA microarray analysis revealed that 335 transcripts were responsive to ^125^I-mediated DNA damage in AG09319 cells in culture, whereas 49 and 27 transcripts were differentially expressed in ^125^I-IUdR-targeted GM05388 and NHFK cells in culture, respectively (Figure 1). Out of 335 identified transcripts in AG09319 cell cultures, 189 belong to named genes (see Additional file 1). The majority of differentially expressed transcripts in AG09319 cell cultures (171 transcript) were repressed in response to ^125^I-mediated DNA damage, whereas 164 transcripts were induced (Figure 1A, B). Among up-regulated genes we found genes involved in cell cycle regulation (*DUSP1*,*FGF2, SHC*, and others) and regulation of cell cycle checkpoints (*CCNG1*,*CDKN1A, GADD45A*, and others). The major mechanism of regulation of cell cycle for identified genes (*CCNG1*, *CDKN1A*, *FGF2*, *GADD45A *and *SHC1*) is via modulation of protein kinase activity driving the cell cycle machinery. *CDKN1A*, *GADD45A *and *CCNG1 *all contribute to cell cycle arrest following genotoxic stress; these genes are also among the best studied and thoroughly validated markers of IR exposure [12,13]. In our study, we observed the up-regulation of several genes (*DUSP1*, *SHC1, PRDX5, TXNRD1, GLRX*, and others) thought to be involved in regulation of cell redox balance [14]. Surprisingly, many of ^125^I-induced genes in AG09319 cells are implicated in formation and regulation of cytoskeleton and extracellular matrix (*TPM2*, *ACTA2*, *TAGLN*, *FN1*, *CALD1*, *TIMP3*, *MMP2*, *KRTAP1-5, ARHE*, and others). This observation raises the issue of a possible interconnection between DNA damage response and likely downstream changes in cell shape and/or motility, the finding that deserves to be addressed more specifically in future. Among 171 down-regulated transcripts that were responsive to ^125^I-mediated damage in AG09319 cells in culture, 88 transcripts belonged to named genes. Of these, 25 genes are implicated in cell cycle regulation, among them 17 genes being responsible for governing M phase transition and/or cytokinesis (*ANLN*, *CCNA2, CCNB1*, *CDC2, CDC2L2*, *CCNB2*, *CDKN2C, CDKN3, CKS1B*, *CKS2*, *KIF11, KIF23*, *KPNA2, PCNA, MAD2L1*, *PRC1*, *PTTG1*, *RAD21*, *SMC4L1, UBE2C *and others). Another large set of repressed genes is involved in DNA metabolism, particularly in DNA replication, DNA repair and/or DNA damage response (*ADPRT, HMGB1*, *HMGB2*, *PCNA*, *PTTG1*, *PTTG2*, *RAD21*, *RPA1*, *RRM1 *and *RRM2*). Several genes belonging to large gene families were down-regulated in common in AG09319 cell cultures; these include insulin-like growth factor binding proteins (*IGFBP3, IGFBP4 *and *IGFBP7*), pregnancy-specific glycoproteins with unknown function (*PSG1, PSG2, PSG3, PSG6, PSG9 *and *PSG11*), minor variants of histones (*H2AFX*, *H2AFV*, *H2AFZ, H3F3B *and *HIST1H4C*) implicated in DNA packaging and members of heterogeneous ribonucleoprotein family (*HNRPA1*, *HNRPA2B1, HNRPF, HNRPH1*, *HNRPD, HNRPR *and *HNRPU*) involved in RNA processing. Interestingly, the phosphorylation of one of the histones identified, namely *H2AFX*, is known to be one of the earliest events in DNA DSBs response [15] and *HNRPK *was shown recently to be the transcriptional co-activator of p53 in DNA damage response [16]. However, whether the coordinated down-regulation of identified gene families at the transcript level serves the regulatory role in DNA damage response is not clear.

We identified 16 ^125^I-responsive differentially expressed genes that were up-regulated in GM05388 and NHFK cell cultures (Figure 1A; and see Additional files 2, 3). The majority of induced genes in GM05388 cells are also induced in AG09319 cells. These include *CDKN1A*, *GADD45A, DKK1, ARHE, FGF2 *and *FHL2*. We found 33 ^125^I-responsive differentially expressed genes that were repressed in GM05388 cells and 11 genes down-regulated in NHFK cell cultures (Figure 1B). *HMGB1, HMGB2, H2AFX, H2AFZ *and *HIST1H4C *are among consistently down-regulated genes in both AG09319 and GM05388 fibroblast cells. In contrast to fibroblast gene expression profiles, the pattern of gene modulation in normal human keratinocytes in response to ^125^I-induced damage is distinct, with only two transcripts being common for keratinocytes and any of fibroblasts tested.

**Figure 2:**
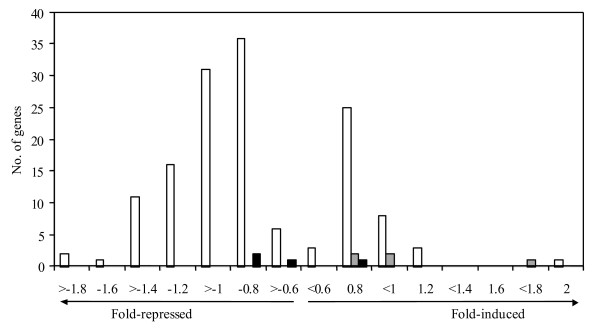
**Log fold-change distribution in the expression level of ^125^I-IUdR-responsive genes in primary human cell lines**. Shown in white – AG09319 cells, in grey – GM05388 cells, in black – NHFK.

We evaluated the magnitude of the changes in the expression level for ^125^I-responsive transcripts by comparing the log fold-change values with the number of modulated genes showing this change in expression (Figure 2; and see Additional files 1, 2, 3). For the majority of differentially expressed transcripts these alterations were less than 2-fold comparing with control in all cell lines examined. For AG09319 cell line, only 34 out of 335 transcripts identified showed more than 2-fold changes in their abundance, and only a few of these transcripts (*CDKN1A*, *KRTAP1-5 *and *ARHE*) were up-regulated. *CDKN1A *was the only gene induced more than 2-fold in GM05388 cells, and no transcripts/genes appeared to be modulated to such an extent in NHFK.

### Effect of increasing concentration of ^125^I-IUdR in cell culture media on gene expression changes

**Figure 3:**
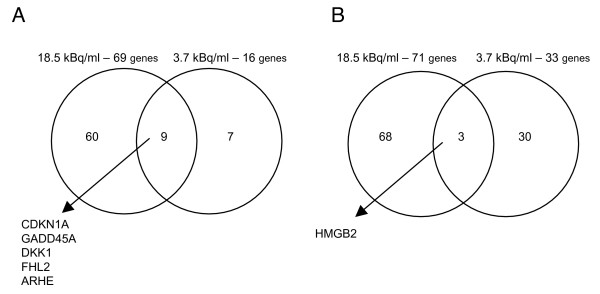
**^125^I-IUdR-responsive sets of either induced (A) or repressed (B) genes in GM05388 human cells**. Different concentration of ^125^IUdR in cell culture media was used.

In the previously described experiments, cells were radiolabeled by incubation in a medium supplemented with 3.7 kBq/ml of ^125^I-IUdR for 24 h followed by incubation in IUdR-free medium for another 24 hours. However, in separate experiments, the effect of 18.5 kBq/ml of ^125^I-IUdR in a media was also evaluated. It was shown previously that there is a linear increase in DNA incorporation of ^125^I-IUdR depending on concentration of this compound in cell culture media [17]. We attempted to address the question of whether the increasing concentration of ^125^I-IUdR in cell culture media qualitatively and/or quantitatively affect gene expression changes in primary human cells. To this end, GM05388 cell line with a lack of robust gene expression changes in response to 3.7 kBq/ml of ^125^I-IUdR was exposed to 5-fold higher concentration of ^125^I-IUdR (see Methods section for a detailed description). Surprisingly, the majority of differentially expressed genes/transcripts modulated by different concentrations of ^125^I-IUdR in cell culture media being induced or repressed by either 3.7 kBq/ml or 18.5 kBq/ml concentrations of ^125^I-IUdR are distinct (Figure 3). Only a few, mainly p53-dependent, genes are modulated in common by both concentrations. Such a non-linearity between the dose of radiation and the effect of differential gene expression was observed recently for cultured human keratinocytes exposed to 0.2 Gy and 4 Gy doses of X-rays [18]. Interestingly, the increase in the number of differentially expressed genes/transcripts modulated by DNA-incorporated ^125^I-IUdR in GM05388 cell line parallels the increase of ^125^I-IUdR concentrations in cell culture media (Figure 3; and see Additional files 2 and 4).

### Classification of differentially expressed genes in functional groups

Classification of genes in functional groups was done on the basis of biological process categories from the Gene Ontology Consortium (GO) [19]. Lists of all differentially expressed genes identified by ANOVA test for each cell line were compared to all annotated genes in the array by using the categorical over-representation function of EASE software [20]. We found that the key up-regulated biological processes in a chosen panel of cell lines are the regulation of protein kinase activity, regulation of cell cycle machinery and/or cell death/apoptosis (Table 1). Genes repressed in response to ^125^I-IUdR treatment are involved in cytokinesis, M phase of the cell cycle, chromosome architecture and organization, DNA metabolism, DNA packaging, DNA repair and response to DNA damage (Table 1). In our studies, ^125^I-IUdR-mediated gene expression profiles demonstrate the apparent induction of genes involved in negative regulation of cell cycle via checkpoint control and down-regulation of genes implicated in cell transition through M phase and/or cytokinesis.

**Table 1:** Functional annotation of selected overrepresented Gene Ontology categories as determined by EASE

	Up-regulation		Down-regulation	
	
Cell Line	Biological processes	EASE score	Biological processes	EASE score
AG09319			Cytokinesis	1.09E-15
	Regulation of enzyme activity	0.0001	Cell cycle	9.68E-13
	Regulation of protein kinase activity	0.0013	M phase	1.95E-12
	Regulation of cell size	0.0038	DNA metabolism	1.62E-07
	Negative regulation of signal		RNA processing	3.32E-06
	transduction	0.0116	Chromosome organization	0.0001
	Regulation of cell proliferation	0.0158	Cytoskeleton organization	0.0002
	Positive regulation of apoptosis	0.0186	DNA replication	0.0002
	Activation of MAPK	0.0236	DNA repair	0.0013
	Cell redox homeostasis	0.0485	Response to DNA damage	0.0021
			DNA packaging	0.0022
			Primary metabolism	0.0083
GM05388 (3.7 kBq//ml)	Regulation of protein kinase activity	0.0014	DNA base excision repair	0.0022
	Regulation of enzyme activity	0.0048	DNA metabolism	0.0134
	Cell cycle arrest	0.0335	DNA packaging	0.0174
	Cell cycle	0.0453	Chromosome organization	0.022
			Cytoskeleton organization	0.0376
GM05388(18.5 kBq/ml)	Cell death	0.0028	M phase	7.63E-06
	G1/S transition of mitotic cell cycle	0.0067	Chromosome organization	0.0004
			Cell cycle	0.0008
			Cytokinesis	0.0058
			DNA packaging	0.0139
			Protein metabolism	0.0430

### Non-random distribution of differentially expressed genes following ^125^I-IUdR labeling of the primary human cell lines

**Figure 4:**
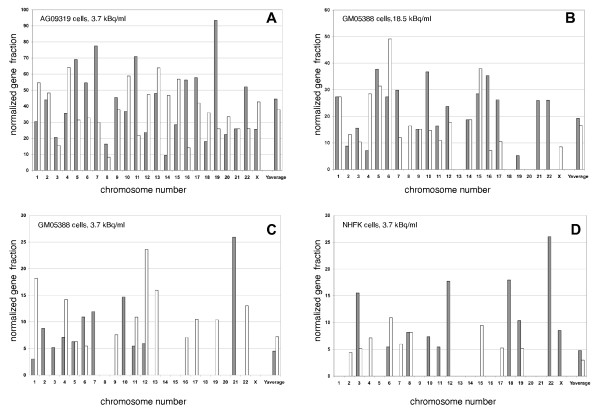
**Non-random chromosomal distribution of differentially expressed genes after ^125^I-IUdR treatment of human cells**. Shown on X axis is the chromosome number, shown on Y axis is the normalized fraction of differentially expressed genes on a given chromosome. Patterned bars in front represent upregulated genes, open bars represent down-regulated genes. **A **– AG09319 cells, **B **– GM05388 cells (18.5 kBq/ml of ^125^IUdR), **C **– GM05388 cells (3.7 kBq/ml of ^125^I-IUdR), **D **– NHFK cells.

Analysis of ^125^I-IUdR-specific gene expression in human cells shows that the incidence of genes either up-regulated or down-regulated following radionuclide labeling varied substantially on different chromosomes and was independent of chromosome length (Figure 4). To investigate a possible relationship between ^125^I-triggered gene expression and gene location, chromosomal positions for probes represented on Agilent Whole Human Genome arrays were retrieved. As shown in Figure 4, a disproportionately high number of up-regulated ^125^I-specific genes were located on chromosomes 19, 7, 5, and 11 (for AG09319 cell line), on chromosomes 5, 10, 16, 7 and 15 (for GM05388 cell line) and on chromosomes 22, 18 and 12 (for NHFK). Similarly, we observed a non-random enrichment of ^125^I-repressed genes on chromosomes 4, 13, 10, 15 and 1 (for AG09319 cells), on chromosomes 6, 15 and 5 (for GM05388) and on chromosomes 6, 15, 8 and 4 (for NHFK). This disproportionality was independent of the density of ascertained genes on these chromosomes. For example, chromosomes 19 and 3 contained a similar number of genes (1,928 and 1,933, respectively), but 4.5 times as many number of genes on chromosome 19 showed up-regulation after ^125^I-IUdR-induced DNA damage in AG09319 cell line.

### Real-time PCR validation of DNA microarray data

Six selected genes were further analyzed by real-time, quantitative PCR to validate the results obtained using DNA microarray (Table 2). These genes (*CDKN1A, GADD45A, IGFBP3, H2AFX, TRIM22 *and *FHL2*) showed different magnitude of either induction or repression following ^125^I-IUdR treatments in different cell lines as determined by our DNA microarray studies. Also, these genes were picked up for additional in-depth study because they are involved in different cellular functions. For each gene, RT-PCR reactions were performed using three replicate independent RNA samples of different cell lines. RT-PCR was done three times for each sample, and the data were averaged and normalized to 18S RNA amount. The relative ratios of transcript amounts in ^125^I-IUdR-treated and ^127^I-IUdR-treated samples were compared with microarray data of the matched input RNA sample by taking into account the mean values and standard errors of measurement representing at least three independent replicate experiments for DNA microarray and RT-PCR studies (Table 2). The results confirmed the similar transcriptional response for all six genes tested with two independent techniques, namely, DNA microarray and RT-PCR assays, thus proving the accuracy and reproducibility of our DNA microarray dataset.

**Table 2:** Comparison of DNA microarray and real-time PCR measurements. Shown are the mean and the standard error of the mean for at least three independent experiments.

		Microarray ratio	RT-PCR ratio
		
Gene symbol	Cell line	Mean ± SEM	Mean ± SEM
CDKN1A	AG09319	4.17 ± 0.75	2.72 ± 0.22
	GM05388	3.45 ± 0.48	2.99 ± 0.49
	NHFK	1.17 ± 0.20	1.17 ± 0.22
GADD45A	AG09319	1.77 ± 0.24	1.37 ± 0.31
	GM05388	1.57 ± 0.35	1.23 ± 0.17
	NHFK	0.90 ± 0.15	0.79 ± 0.18
IGFBP3	AG09319	1.59 ± 0.11	1.06 ± 0.13
	GM05388	1.15 ± 0.22	1.10 ± 0.17
	NHFK	1.16 ± 0.06	0.74 ± 0.41
H2AFX	AG09319	0.41 ± 0.05	0.18 ± 0.06
	GM05388	0.62 ± 0.13	0.41 ± 0.15
	NHFK	0.80 ± 0.21	0.70 ± 0.21
FHL2	AG09319	1.79 ± 0.31	0.97 ± 0.11
	GM05388	1.75 ± 0.46	1.15 ± 0.17
	NHFK	1.16 ± 0.40	1.03 ± 0.27
TRIM22	AG09319	1.38 ± 0.17	1.07 ± 0.17
	GM05388	1.95 ± 0.41	1.63 ± 0.13
	NHFK	1.82 ± 0.54	2.22 ± 1.20

## Discussion

The primary goal of our study was to characterize the response of primary human cells to irradiation from DNA-incorporated radionuclide in a form of the thymidine analog 5- [^125^I]iodo-2'-deoxyuridine (^125^I-IUdR) in terms of gene expression changes. To date, there was only one report addressing the effect of a cell-incorporated radionuclide (^35^S) on gene expression changes in human colorectal carcinoma cells [21]. That study showed that ^35^S-methionine induces a much more robust transcriptional response than gamma-radiation exposure. But this limited information was available only for a few thousand of human genes. Our study is the first to use a whole-genome DNA microarray platform covering all known and predicted genes to assess the effect of cell-incorporated radionuclide (^125^I) on transcriptional response in primary human cells. It is well-known that decay of DNA-incorporated ^125^I induces mainly DNA double-strand breaks (DSBs) while producing little damage to the rest of the cell [17,22]. That the transcriptional responses to ionizing radiation administered as external beam irradiation vary widely in cell lines with different tissues of origin and different genetic backgrounds was shown in previous studies [12]. Our study is the first to demonstrate the heterogeneity of normal human cell responses to internal irradiation, highlighting the importance of cellular context for reaction to DNA DSB formation. Only a few ^125^I-IUdR-responsive genes, mainly showing p53-dependent regulation, are modulated in common in the several human cell lines we tested. In our previous study we found that expression of 206 genes was altered in normal human lung IMR-90 fibroblasts, with a distinct transcriptional profile sharing only a few genes in common with AG09319 and GM05388 cells used in the present study [7]. This, in fact, is consistent with the published data showing a surprisingly little overlap for genes differentially expressed in various cell lines following external beam IR exposure in the same dose [12,23]. For example, it was shown recently that only 13 genes out of 463 genes up-regulated by more than 2-fold following IR exposure in 2 Gy were shared by three different cell lines used [23]. The observed lack of consensus gene expression changes in normal human cells in response to internal irradiation can be explained by the recent finding showing that translational level of gene expression regulation following genotoxic stress might be crucial [24]. In addition, since the cell lines used in our study represented distinct differentiated cell types and/or tissues of origin, specific transcriptional programmes are likely to be operative in these cells favoring expression of unique sets of genes which might be distinct for each cell line. This assumption is reinforced by the observation that two fibroblast cell lines that we used in the present study shared 35 differentially expressed genes compared to only 3 genes that were common to keratinocytes as well (Figure 1); thus, fibroblasts of different tissues of origin are being more closely related to each other than to keratinocytes with regard to ^125^I-IUdR-driven changes in gene expression.

In contrast to the majority of microarray-based papers published so far addressing the gene expression changes in human cells in response to external gamma-radiation exposure, we found that the limited subset of genes is differentially expressed in normal ^125^I-IUdR-labeled, primary human cells. This finding implies that DNA DSB induced by ^125^I decay in human cells that is believed to be hard to repair is not able to elicit robust transcriptional response regardless the well-known deleterious effect of DNA-incorporated ^125^I on cell survival. Interestingly, that there is no apparent relationship between the genes necessary for survival from the DNA-damaging agents and those genes whose transcription is increased after genotoxic stress exposure is already shown for yeast [25]; in light of our findings, this may be true for human cells as well. This assumption is reinforced by our observation that by the time when ^125^I-IUdR-induced gene expression alterations were examined in primary human cells, no significant changes in cell viability occurred as judged by trypan blue cell exclusion assay with more than 90% of cells being viable.

We found that a significant fraction of induced genes in fibroblast cell lines are p53-dependent in their transcriptional regulation. This set of genes include established p53-targets like *CDKN1A*, *GADD45A*, *CCNG*, *PLAB*, *DUSP1*, *FHL2*, *SERPINE1*, *IGFBP3*, *DKK1 *and also predicted targets of TP53 like *IGFBP4*, *ANXA4*, *FGF2*, *ACTA2*, *FN1*, *CALD1 *and *TIMP3 *[26]. As was the case for induced genes, many of repressed genes found are known to be regulated by p53, for example *PCNA*, *PTTG1*, *CCNB1*, *PRC1*, *RRM2*, *CCNB2*, *CDKN2C*, *PTMA*, *KPNA2*, *MAD2L1*, *CBX1 *and *HMGB2 *[26]. However, the fraction of these verified and predicted targets of p53 in repressed set of genes is considerably smaller than that for up-regulated genes.

External IR exposure can induce the transcription of specific genes through the activation of not only p53 but also NF-κB and AP-1 [27]. External gamma-irradiation results in damage to the subcellular constituents distributed more or less uniformly across the entire target cell volume. Using external gamma-radiation as a genotoxic stress agent, expression level modulation of thousands of genes in normal human cells was reported recently [3]. In contrast, our study shows that DNA-targeted IR-induced damage elicited by ^125^I-IUdR results in an order of magnitude less robust transcriptional response. That only the limited subset of genes, many of which are known components of p53-signaling network, is implicated in response to ^125^I-IUdR-triggered DNA damage may be the consequence of the limited repertoire of the sensing pathways involved in detection of such damage. Indeed, the key proteins of DNA DSBs signaling such as ATM and components of MRN complex are localized in the nucleus that is the target for ^125^I-mediated damage. ATM is known to be activated by just a few DNA DSBs following IR exposure [28], that, in turn, triggers the plethora of downstream ATM-dependent responses including the accumulation of p53 and increased binding affinity of p53 to transcriptional targets [29]. Thus, the activation and/or repression of downstream p53 target genes are the consequences of ^125^I-mediated DNA damage.

In contrast, NF-κB and AP-1 normally reside in the cytoplasm in the inactive state requiring oxidative conditions in the cytoplasm for their activation [30]. The decays of DNA-incorporated ^125^I produce little damage to the cytoplasm of treated cells that is likely to be insufficient for the activation of these transcription factors thus explaining the prominent role of p53 signaling network following DNA-targeted damage. In support of this view, the increase of ^125^I-IUdR incorporation into DNA in GM05388 cells in our studies parallels the increase in the number of differentially expressed genes. Provided that following decay of DNA-incorporated ^125^I the energy deposition in cytoplasm is less than 5 % of that in the nucleus, it is likely that the increased number of decays of DNA-incorporated ^125^I results in oxidative stress condition in cytoplasm giving rise to activation of some additional signaling pathways as well. Interestingly, the cellular responses to "high" (18.5 kBq/ml) and "low" (3.7 kBq/ml) concentrations of ^125^I-IUdR in cell culture media were so apparently distinct, that they resemble the recent finding of putative exclusive "low-dose" and "high-dose" gene responders following either IR exposure of cultured normal human cells or irradiation of the whole mice in different doses [18,31,32]. Indeed, in an effort to develop gene expression signatures that predict IR exposure in mice and humans, only 5 genes out of total 225 genes found to be modulated in peripheral blood cells in mice exposed to increasing doses of either 0.5 Gy, 2 Gy or 10 Gy of IR were common to all these exposures [32]. What emerges from these studies is that there is no "clear-cut" dose-response effect for the vast majority of IR-induced genes and that distinct biological processes are modulated as a function of IR dose. In our present study, we found that only a few, mainly p53-dependent, genes like *CDKN1A*, *GADD45A*, *DKK1*, *FHL2 *are modulated in common by two various concentrations of ^125^I-IUdR. Interestingly, the most robust transcriptional response among the cell lines we used was seen for AG09319 fibroblasts which, by our estimation, accumulated almost two times more DNA DSBs compared to either GM05388 or NHFK cells (see Methods section). The increase in the number of modulated genes also seems to be dependent on the amount of ^125^I-IUdR as the higher (18.5 kBq/ml) concentration of ^125^I-IUdR in cell culture media apparently results in the larger number of genes being modulated by internal IR. This might, at least, in part explain why the number of genes responding to ^125^I-IUdR treatment was very different within the three cell lines. However, whether the threshold for modulation of gene expression exists in case of ^125^I-IUdR-triggered DNA damage is presently not clear.

By calculating the ratios of relative gene expression changes following either ^125^I-IUdR or ^127^I-IUdR cell culture treatment, we have determined the magnitude of transcriptional response to ^125^I-elicited DNA damage in normal human cells. As indicated in our results (Figure 2), only a few genes/transcripts changed their expression level more than twofold. Such small changes at a global level after IR exposures were reported previously by several groups, both in human cell fibroblast and mouse studies [18,33]. The minimal fold change in the set of genes identified by ANOVA as being differentially expressed in our study was 1.35 – 1.4, which is close to a cutoff value used in a recent paper [31]. The biological significance of such a change depends on the particular gene under consideration. However, we have achieved a good cohort of DNA microarray and real-time PCR datasets that closely parallel each other even when the changes in gene expression are small. This agreement between two independent techniques used for gene expression studies implies that the small changes underlying the cellular response to ^125^I-triggered DNA damage we observed in our experiments are significant and reliable.

Our results suggest that the cell cycle-related processes dominate over all other ^125^I-perturbed pathways at the transcriptome level. These findings may be explained by the fact that almost all ^125^I-IUdR is incorporated in cellular DNA during S-phase. DNA DSBs formation throughout the G2 phase is likely to activate one of the key components of the DNA damage response, namely p53 [34]. p53 can act as an effector in inducing the G2/M block through the induction of *CDKN1A*, *GADD45A*, *CCNG1 *and the repression of *CCNB1 *and *CCNB2*. Also, p53 was recently mechanistically linked to the cytokinesis providing the negative feedback for this process [35]. Although the gene expression profile characteristic for ^125^I-triggered DNA damage clearly suggest the ongoing cell cycle arrest in targeted cell cultures, it is unknown whether this halt in cell proliferation is reversible or not. Interestingly, a subset of the ^125^I-responsive genes is represented by known cellular senescence markers. These include up-regulated *SERPINE1*, *SERPINE2*, *CDKN1A*, *SHC1*, *IGFBP3*, *IGFBP7*, *SPUVE*, *CITED2*, *S100A4*, *FN1*, *PSG1*, *CALD1*, *TAGLN*, *GLRX*, *LOXL2*, *ATP6V0C*, *SPARC*, *DKK1*, *DKK3*, *CAP2*, *CTGF *as well as down-regulated *PPP1CC*, *H3F3B*, *CDC2*, *RBBP7*, *HNRPH1*, *PTMA *and *CCNA2 *in AG09319 cells; induced *CDKN1A*, *PERP*, *RPS27L*, *FST *and *DKK1 *as well as repressed *CCNA2*, *EIF3S6 *and *HNRPF *in GM05388 cells; and *TM7SF3 *in NHFK cells. This suggests that the cellular senescence is likely be triggered in response to DNA damage elicited by ^125^I in at least fibroblast cell lines we tested [36].

Chromosomal clustering of genes whose expression was up-regulated during replicative senescence was recently observed in primary human fibroblast and epithelial cells [36]. Since we identified many senescence-related genes as being differentially expressed following ^125^I-triggered DNA damage in our studies, we wished to examine the chromosomal distribution of induced and repressed genes more closely. That the differentially expressed ^125^I-responsive genes reveal non-random distribution across distinct chromosomes was a surprising finding that was never reported in the literature for radiation-induced gene profiles before. ^125^I-IUdR is incorporated into cellular DNA at S phase of the cell cycle presumably in a random order; the non-random chromosomal distribution of differentially expressed genes cannot be explained by the fact that ^125^I-IUdR had incorporated predominantly to some specific chromosomes since we grew our asynchronous cell cultures in a medium supplemented with ^125^I-IUdR for 24 hrs that is approximately the population doubling time for all cell lines we used. A follow-up analysis of our earlier results on DNA microarray studies of gene expression changes in normal human fibroblasts after both high-dose rate and low-dose rate external beam gamma-radiation exposures [7] revealed random distribution of induced genes across different chromosomes, even if only a random subset of genes comparable in size to that observed following ^125^I-IUdR exposure, was examined (data not shown). Interestingly, the non-randomness observed in our present study was found to be a characteristic of not only the induced but also the down-regulated genes which is in contrast to earlier evidence on replicative senescence [36]. This apparent discrepancy is likely to be explained by the different triggers of senescence in these cases – e.g. shortening of telomeres resulting in the replicative senescence or ^125^I-induced DNA damage possibly leading to premature stress-induced senescence. Previous data that histone deacetylase inhibitors, that decondense chromatin, induce a senescence-like state in human fibroblasts suggested that the opening of certain chromatin domains (for example, conversion of heterochromatin to euchromatin) may be a feature of replicative senescence [37]. Following ^125^I-IUdR treatment of cells we found extensive down-regulation of many genes whose products are involved in chromatin condensation (such as *HCAP-G *and eight distinct members of histone H1 family in GM05388 cells). It is possible that highly localized DNA damage elicited by ^125^I decay also leads to the relaxation of higher-order chromatin structures in the vicinity of the site of decay and to the opening of some chromatin domains thus recapitulating some features of histone deacetylase inhibitor-induced premature senescence.

It is known that DNA DSBs are intentionally formed in human cells under some strictly controlled conditions, for example in meiosis and in VDJ recombination. Recent evidence suggest that a topoisomerase IIβ-mediated DNA DSB is normally required for regulated transcription [38] as well. Upon binding of nuclear receptors to the target gene, Topoisomerase IIβ creates a nucleosome-specific DNA double-strand break that triggers PARP-1's intrinsic catalytic activity, resulting in poly(ADP-ribosyl)ation of chromatin-associated proteins. This leads to the exchange of histone H1 for high mobility group B (HMGB) proteins during this gene-specific transcriptional activation [38]. It was shown that topoisomerase IIβ-mediated DNA DSB is required for efficient transcription initiation for at least five different promoters; therefore, it is highly likely that this phenomena is general. The emerging view that the transcription is far more widespread in human cells than it was thought before suggests that all of the non-repeat portions of the human genome are transcribed [39]. Even though ^125^I-IUdR is incorporated into cellular DNA at random sites, given the estimates that 90% of the human genome are transcribed [40] makes it reasonable to assume that some ^125^I decays may occur within promoter regions. Interestingly, we observed strong down-regulation of HMGB genes (*HMGB1 *and *HMGB2*) upon ^125^I-IUdR labeling in all fibroblast cell lines we used. PARP-1 was also repressed in GM05388 cells. These findings raise the possibility that fibroblasts can somehow distinguish Topoisomerase IIβ-induced "physiological" DNA DSBs from ^125^I-IUdR-induced DNA DSBs; the resulting alteration in cellular signaling may lead to regulated repression of the components of machinery that normally helps DNA transcription to start, at least for some gene targets.

The comparison of gene expression profiles following exposure of human cells to the same compound, labeled either with "hot" or "cold" iodine, allowed us to rule out all possible side effects of addition of ^125^I-IUdR into the cell culture media other than direct genotoxicity. Such "clear-cut" discrimination of genotoxic mode of action of the drug in gene expression studies was done, to the best of our knowledge, for the first time. The results of our present study imply that DNA-targeted ^125^I-triggered damage appears to modulate the gene expression changes of a limited subset of genes in primary human cells. This may prompt to reconsider the findings of the previously published experiments on genomic profiling of human cells following IR exposures in that the "DNA damage responsive" subsets of genes identified perhaps should be regarded as "IR responsive" [3,41,42]. In practical terms, the biological deleterious effects stemming from DNA-targeted IR-induced damage such as that induced by DNA-incorporated ^125^I is easily explained at the molecular level, given the persistence of DNA damage in cell cultures following continuous accumulation of ^125^I decays, the repression of several major components of DNA repair machinery and the lack of robust response to DNA-targeted IR damage at least at the transcriptome level observed in our study.

## Conclusion

Our data suggest that DNA-targeted ionizing radiation produced by ^125^I-IUdR results in changes in expression of only a limited subset of genes in primary human cells. The responsive genes are distributed non-randomly among the chromosomes; and a significant fraction of them is p53-dependent in the transcriptional regulation.

## Methods

### Tissue culture

Human primary cell lines of different origin, namely, gingival fibroblasts AG09319 (population doubling level, PDL 22) and fetal skin fibroblasts GM05388 (PDL 21; both from Coriell Cell Repositories) were grown in Eagle's minimum essential medium (EMEM; ATCC) supplemented with 10% FBS, non-essential amino acids, 1 mM sodium pyruvate and 2 mM L-glutamine in a humidified 5% CO_2 _incubator at 37°C. Neonatal foreskin epidermal keratinocytes (NHFK, PDL 19) pooled from several donors' explants (Coriell Cell Repositories) were cultivated in Keratinocyte-SFM (Invitrogen) supplemented with L-glutamine, epidermal growth factor (0.2 ng/ml) and bovine pituitary extract (30 μg/ml) under the same conditions. Since it is known that primary normal human cells have a finite lifespan in culture, the choice of cell lines used in this study was dictated by the availability of primary human cells with the lowest passage number attained in culture provided by the Coriell Cell Repositories.

### ^125^I-IUdR radiolabeling

Iodine-125 labeled iododeoxyuridine (specific activity ~74 TBq/mmol) was purchased from MP Biomedicals. The desired radioactive concentration was obtained by dilution of the ^125^I-IUdR in fully supplemented EMEM. On a day before radiolabeling, the cells were subcultured so that they reached approximately 50% confluence on the next day. The cell cultures were divided into two batches. One batch was radiolabeled by cell incubation in a medium supplemented either with 3.7 kBq/ml of ^125^I-IUdR or with 18.5 kBq/ml of ^125^I-IUdR (GM05388 cells) for 24 h followed by incubation in IUdR-free medium for another 24 hours. The second batch of cell cultures, served as a control, was incubated under identical conditions except that ^125^I-IUdR was substituted for the equimolar concentration of non-radioactive ^127^I-IUdR (Sigma). Each experiment was carried out independently four times. Given that asynchronous cell populations were used for ^125^I-IUdR treatment, we chose to perform cell radiolabeling over 24 hours which approximately corresponds to duration of one cell cycle; therefore, all cycling cells were destined to incorporate ^125^I-IUdR and be exposed to internal IR. None of these treatments had a marked effect on viability of cell cultures by the end of the exposure as measured by the trypan blue exclusion assay; nuclei integrity was maintained in ^125^I-IUdR-treated cells providing no evidence for apoptosis as observed by DAPI nuclei staining (data not shown).

### ^125^I-IUdR uptake studies

A separate experiment was run in parallel with that described above. Following incubation in either ^125^I-IUdR or ^127^I-IUdR-containing media according to described protocol (see ^125^I-IUdR Radiolabeling), the labeled cells were trypsinized and counted; the cell pellets were washed in phosphate buffered saline (PBS; Invitrogen) and the DNA-incorporated activity in known numbers of cells was determined following trichloroacetic acid (TCA; Sigma) precipitation [8] using a γ -counter (Biotraces).

### RNA sample preparation, probe labeling and DNA microarray procedure

Total RNA was extracted using Trizol (Invitrogen), and then purified sequentially with RNeasy kit (Qiagen) and TURBO DNA-free kit (Ambion) per instructions of the suppliers. The amount and quality of RNA preparations were evaluated on the Agilent 2100 Bioanalyzer with RNA 6000 Nano Reagents and Supplies (Agilent). Subsequently, cDNA targets were synthesized from 20 μg of total RNA in each reaction and fluorescently labeled with either Cy3-dCTP or Cy5-dCTP using the Agilent Fluorescent Direct Label kit. Then, the cDNA targets corresponding both to ^125^I-IUdR- or ^127^I-IUdR-labeled samples per each cell line were combined and hybridized to 44,290-element Agilent Human Whole Genome oligo microarrays using Agilent SureHyb hybridization chambers; methods for microarray hybridization and washing were as described in manufacturer's protocol. Hybridized DNA microarrays were scanned with a resolution of 5 μm on a VersArray ChipReader scanner (Bio-Rad, Inc.), and TIFF images were processed by VersArray Analyzer 4.5 software (Media Cybernetics, Inc.). All samples had four independent biological replicates, and each replicate was run on separate slides.

### Data analysis

Raw intensity profiles were analyzed using BRB-ArrayTools Version 3.2.2 software developed by Dr. Richard Simon and Amy Peng Lam (Biometric Research Branch, National Cancer Institute, NIH). The input for this analysis included ^127^I-IUdR-treatment versus ^125^I-IUdR-treatment data to determine the ^125^I-responsive genes from four independent experiments per each cell line. Differentially expressed genes were identified using a random variance ANOVA test. Changes in gene expression were considered statistically significant if the p values for corresponding genes were less than 0.005. The false discovery rate (FDR) was controlled to be less than 10%. The resultant sets of differentially expressed genes were further analyzed for functional significance using the program EASE (version 2.0). This software obtains the Gene Ontology (GO) annotations from a database and generates a statistical analysis of the functional annotations that are overrepresented in the inputted list of genes [20], with a Bonferroni correction for multiple comparisons included. GO biological processes with EASE scores less than 0.05 were considered to be statistically significant [43]. The analysis of the distribution of differentially expressed genes on different chromosomes was done with the help of BRB-ArrayTools Version 3.2.2 software, including the assignment of all spotted and differentially expressed genes to specific chromosomes. The normalized fraction of differentially expressed genes is calculated based on the relative enrichment of these modulated genes on a given chromosome.

Minimum Information About a Microarray Experiment (MIAME)-compliant raw data for this series of experiments have been deposited in the ArrayExpress database maintained by the European Bioinformatics Institute (accession no. E-MEXP-929) [44].

### Quantitative real-time PCR

In total, six genes were chosen for RT-PCR validation. The RT-PCR was performed on three replicate independent RNA samples for each cell line. The complementary DNA was synthesized from total RNA by reaction with MultiScribe reverse transcriptase and random primers (both from High Capacity cDNA Archive kit; Applied Biosystems) according to the manufacturer's protocol. For each gene, PCR reactions were run three times on one sample. PCR was performed on iCycler iQ (Bio-Rad, Inc.) in 20-μl reactions by using TaqMan Assay-on-Demand primers/probe sets (Applied Biosystems). IDs of the TaqMan Assay-on-Demand primers/probe sets used are as follows: Hs00355782_m1 (*CDKN1A*), Hs00169255_m1 (*GADD45A*), Hs00181211_m1 (*IGFBP3*), Hs00266783_s1 (*H2AFX*), Hs00179935_m1 (*FHL2*) and Hs00232319_m1 (*TRIM22*). The reaction was repeated for 40 cycles; each cycle consisted of denaturing at 95°C for 15 s, annealing and synthesis at 60°C for 1 min as per manufacturer's instructions. Real-time PCR data were analyzed using the comparative CT method within the log-linear phase of the amplification curve obtained for each primers/probe set [13]. The relative amounts of transcript of the tested genes were normalized by 18S rRNA endogenous control primers/probe set. The average ratios of relative amounts of transcript in ^125^I-IUdR-treated versus ^127^I-IUdR-treated cells from three replicate runs were calculated.

## Authors' contributions

MVS participated in the design of the study, conducted all experimental procedures including tissue culture, ^125^I-IUdR radiolabeling and uptake studies, did array experiments, data mining, statistical analysis, verified results with quantitative RT-PCR and drafted the manuscript. RDN conceived of the study and helped to draft the manuscript. IGP participated in the design of the study, drafted the manuscript and edited visual appearance. All authors read and approved the final manuscript.

## Supplementary Material

Additional file 1 ^125^IUdR – responsive set of genes in AG09319 cell line Click here for file

Additional file 2 ^125^IUdR – responsive set of genes in GM05388 cell line (3.7 kBq/ml) Click here for file

Additional file 3 ^125^IUdR – responsive set of genes in NHFK cells Click here for file

Additional file 4 ^125^IUdR – responsive set of genes in GM05388 cell line (18.5 kBq/ml) Click here for file
