# Supplementary material for: Effects of DNA-targeted ionizing radiation produced by 5-[125I]iodo-2'-deoxyuridine on global gene expression in primary human cells
Source: BMC Genomics. 2007 Jun 26;8:192. doi: 10.1186/1471-2164-8-192 (PMC1934370; doi:10.1186/1471-2164-8-192)
Supplement: Additional file 1 — 125IUdR – responsive set of genes in AG09319 cell line [file 1471-2164-8-192-S1.pdf]

Supplementary table 1. ^{125}IUdR - responsive set of genes in AG09319 cell line

164 Up-regulated Significant Genes (ANOVA, p-value less than 0.005)

NN	Description	GB accession	Gene symbol	Parametric p-value	Log-fold change (^{125}IUdR/^{127}IUdR)
1	Homo sapiens cyclin-dependent kinase inhibitor 1A (p21, Cip1) (CDKN1A), transcript variant 1, mRNA	NM_000389	CDKN1A	<0.0001	1.9957
2	Homo sapiens keratin associated protein 1-5 (KRTAP1-5), mRNA	NM_031957	KRTAP1-5	<0.0001	1.1624
3	Homo sapiens KIAA1199 (KIAA1199) mRNA, partial cds	AY007811	AY007811	0.0001	1.1473
4	Homo sapiens ras homolog gene family, member E (ARHE), mRNA	NM_005168	ARHE	0.0002	1.0557
5	Homo sapiens cDNA FLJ40353 fis, clone TESTI2033520, weakly similar to BILIARY GLYCOPROTEIN 1 PRECURSOR	AK097672	AK097672	0.0001	0.9806
6	Homo sapiens prostate differentiation factor (PLAB), mRNA	NM_004864	PLAB	<0.0001	0.952
7	Unknown	THC1439475	THC1439475	0.0011	0.9411
8	insulin-like growth factor binding protein 3 {3 region} [human, tuberous sclerosis cells, mRNA Partial, 704 nt]	S56205	S56205	<0.0001	0.902
9	Homo sapiens fibroblast growth factor 2 (basic) (FGF2), mRNA	NM_002006	FGF2	0.0002	0.8925
10	Homo sapiens dual specificity phosphatase 1 (DUSP1), mRNA	NM_004417	DUSP1	0.0002	0.8805
11	Homo sapiens serine (or cysteine) proteinase inhibitor, clade E (nexin, plasminogen activator inhibitor type 1), member 1 (SERPINE1), mRNA	NM_000602	SERPINE1	<0.0001	0.8504
12	Homo sapiens leucine proline-enriched proteoglycan (leprecan) 1 (LEPRE1), mRNA	NM_022356	LEPRE1	0.0001	0.8333
13	Plasminogen activator inhibitor 1, a member of the serpin family of serine proteases and inhibitors, plays a role in regulating blood coagulation by inhibiting fibrinolysis, contributes to tumor progression and is a risk factor for cardiovascular ...	I_929648	I_929648	0.0001	0.8225
14	Homo sapiens serine (or cysteine) proteinase inhibitor, clade E (nexin, plasminogen activator inhibitor type 1), member 2 (SERPINE2), mRNA	NM_006216	SERPINE2	0.0005	0.7852
15	Homo sapiens growth arrest and DNA-damage-inducible, alpha (GADD45A), mRNA	NM_001924	GADD45A	0.0007	0.7751
16	AUTO: Protein of unknown function	I_1847938	I_1847938	0.0015	0.7641
17	Homo sapiens four and a half LIM domains 2 (FHL2), mRNA	NM_001450	FHL2	0.0002	0.7633
18	Homo sapiens suppressor of white apricot homolog 2 (SWAP2), mRNA	NM_007056	SFRS16	0.0001	0.74
19	Homo sapiens hypothetical protein MGC35578 (MGC35578), mRNA	NM_153337	SLIC1	0.0036	0.7349
20	Protein with high similarity to pregnancy specific beta-1-glycoprotein 1 (human PSG1), which may modulate the immune system, contains an immunoglobulin (Ig) domain, which may be involved in protein-protein and protein-ligand interactions	I_3591458	I_3591458	0.0004	0.7329
21	Human fibronectin (FN1) mRNA, splice variant, partial cds	U42594	FN1	0.0003	0.7321
22	Human pregnancy-specific beta-1-glycoprotein (SP1) mRNA, complete cds, clone hPS2	M31126	PSG9	0.0016	0.7312
23	Homo sapiens tissue inhibitor of metalloproteinase 3 (Sorsby fundus dystrophy, pseudoinflammatory) (TIMP3), mRNA	NM_000362	TIMP3	0.0007	0.7271
24	Homo sapiens coatomer protein complex, subunit zeta 2 (COPZ2), mRNA	NM_016429	COPZ2	0.0005	0.726
25	Homo sapiens pregnancy specific beta-1-glycoprotein 1 (PSG1), mRNA	NM_006905	PSG1	0.0012	0.7253
26	Homo sapiens caldesmon 1 (CALD1), transcript variant 4, mRNA	NM_033139	CALD1	0.0002	0.7112
27	Homo sapiens p8 protein (candidate of metastasis 1) (P8), mRNA	NM_012385	P8	0.0004	0.7098
28	Homo sapiens insulin-like growth factor binding protein 4 (IGFBP4), mRNA	NM_001552	IGFBP4	0.0005	0.7064
29	Homo sapiens P/OKcl.13 mRNA for mitogen-activated protein kinase kinase kinase, partial cds	AB044546	AB044546	0.0005	0.6994
30	Human pregnancy-specific glycoprotein beta-1 (SP1) mRNA, last exon	M30629	M30629	0.002	0.6987
31	Homo sapiens transgelin (TAGLN), mRNA	NM_003186	TAGLN	0.0005	0.6982
32	Homo sapiens glutaredoxin (thioltransferase) (GLRX), mRNA	NM_002064	GLRX	0.0005	0.6902
33	Homo sapiens cDNA FLJ11812 fis, clone HEMBA1006364	AK021874	AK021874	0.0007	0.6846
34	Homo sapiens pregnancy specific beta-1-glycoprotein 6 (PSG6), mRNA	NM_002782	PSG6	0.0018	0.6817
35	Homo sapiens sema domain, immunoglobulin domain (Ig), short basic domain, secreted, (semaphorin) 3C (SEMA3C), mRNA	NM_006379	SEMA3C	0.0011	0.6804
36	Homo sapiens thrombospondin 2 (THBS2), mRNA	NM_003247	THBS2	0.0027	0.6804
37	Homo sapiens cDNA FLJ38671 fis, clone HSYRA2000332, highly similar to Human elastin gene	AK095990	AK095990	0.0005	0.6784
38	Homo sapiens SHC (Src homology 2 domain containing) transforming protein 1 (SHC1), mRNA	NM_003029	SHC1	0.0008	0.6753
39	AUTO: Strong similarity to (Homo sapiens) ZNF216: Protein with homology to zinc-finger proteins, expressed in the cochlea	I_1847739	I_1847739	0.001	0.6751
40	Homo sapiens transgelin, mRNA (cDNA clone IMAGE:4065231), complete cds	BC010946	TAGLN	0.0008	0.6643
41	Homo sapiens annexin A4 (ANXA4), mRNA	NM_001153	ANXA4	0.0004	0.6639
42	Homo sapiens mRNA full length insert cDNA clone EUROIMAGE 1913076	AL359062	AL359062	0.0024	0.6604
43	Homo sapiens insulin-like growth factor binding protein 3, mRNA (cDNA clone MGC:2305 IMAGE:3506666), complete cds	BC000013	IGFBP3	0.0005	0.6579
44	Homo sapiens zinc finger protein 216, mRNA (cDNA clone MGC:10350 IMAGE:3945047), complete cds	BC027707	ZNF216	0.0011	0.6506
45	H.sapiens tropomyosin isoform mRNA, complete CDS	Z24727	Z24727	0.0017	0.6475
46	Homo sapiens insulin-like growth factor binding protein 7 (IGFBP7), mRNA	NM_001553	IGFBP7	0.0012	0.6474
47	Homo sapiens pregnancy specific beta-1-glycoprotein 3 (PSG3), mRNA	NM_021016	PSG3	0.0047	0.6456
48	Homo sapiens cyclin G1 (CCNG1), mRNA	NM_004060	CCNG1	0.0007	0.6408
49	Homo sapiens pp9974 mRNA, complete cds	AF318382	AF318382	0.0007	0.6402
50	Homo sapiens cDNA PSEC0048 fis, clone NT2RP2000028, highly similar to Homo sapiens serine protease mRNA	AK075362	AK075362	0.0032	0.6389
51	Homo sapiens mRNA for FLJ00047 protein, partial cds	AK024455	AK024455	0.0014	0.6378
52	Homo sapiens cDNA FLJ11490 fis, clone HEMBA1001918	AK021552	AK021552	0.0013	0.6324
53	Homo sapiens pregnancy specific beta-1-glycoprotein 11, mRNA (cDNA clone MGC:22484 IMAGE:4732286), complete cds	BC020711	PSG11	0.0028	0.6303
54	Unknown	THC1591470	THC1591470	0.0012	0.628
55	Homo sapiens hypothetical protein MGC10120 (MGC10120), mRNA	NM_173809	MGC10120	0.0019	0.6197
56	Homo sapiens cDNA clone IMAGE:5504902, partial cds	BC040043	BC040043	0.002	0.6179
57	Homo sapiens laminin, gamma 1 (formerly LAMB2) (LAMC1), mRNA	NM_002293	LAMC1	0.0011	0.6167
58	Homo sapiens actin, alpha 2, smooth muscle, aorta (ACTA2), mRNA	NM_001613	ACTA2	0.0008	0.6154
59	Insulin-like growth factor binding protein 7, functions in the regulation of cell proliferation and cell adhesion, may act as a tumor suppressor, may play a role in angiogenesis and in senescence	I_1152228	I_1152228	0.0026	0.6153
60	Homo sapiens glutaminase (GLS), mRNA	NM_014905	GLS	0.0035	0.614
61	Homo sapiens CAP, adenylate cyclase-associated protein, 2 (yeast) (CAP2), mRNA	NM_006366	CAP2	0.0011	0.6113
62	Homo sapiens PAPS synthetase-2 (PAPSS2) mRNA, complete cds	AF074331	AF074331	0.0023	0.609
63	Homo sapiens peroxiredoxin 5 (PRDX5), transcript variant 1, nuclear gene encoding mitochondrial protein, mRNA	NM_012094	PRDX5	0.0017	0.6083
64	Homo sapiens connective tissue growth factor (CTGF), mRNA	NM_001901	CTGF	0.0009	0.608
65	Homo sapiens suppression of tumorigenicity 14 (colon carcinoma, matriptase, epithin) (ST14), mRNA	NM_021978	ST14	0.0021	0.6047
66	Homo sapiens tropomyosin 2 (beta) (TPM2), mRNA	NM_003289	TPM2	0.0005	0.6042
67	Homo sapiens calcium-sensing receptor (hypocalciuric hypercalcemia 1, severe neonatal hyperparathyroidism) (CASR), mRNA	NM_000388	CASR	0.0006	0.6011
68	Homo sapiens likely ortholog of mouse synembryn (RIC-8), mRNA	NM_021932	RIC-8	0.0006	0.5955
69	Homo sapiens pregnancy specific beta-1-glycoprotein 2 (PSG2), mRNA	NM_031246	PSG2	0.0029	0.5947
70	Homo sapiens mRNA; cDNA DKFZp434A0530 (from clone DKFZp434A0530); complete cds	AL136842	CDC42EP3	0.0011	0.5944
71	Homo sapiens protease, serine, 23 (SPUVE), mRNA	NM_007173	SPUVE	0.0028	0.5944
72	Homo sapiens ubiquitin-conjugating enzyme E2 variant 1 (UBE2V1), transcript variant 1, mRNA	NM_021988	UBE2V1	0.0012	0.5927
73	Unknown	ENST00000332989	ENST00000332989	0.003	0.5923
74	Homo sapiens lysyl oxidase-like 2 (LOXL2), mRNA	NM_002318	LOXL2	0.0031	0.5881
75	Homo sapiens chromobox homolog 6 (CBX6), mRNA	NM_014292	CBX6	0.0008	0.5875
76	Homo sapiens Rab acceptor 1 (prenylated) (RABAC1), mRNA	NM_006423	RABAC1	0.0014	0.5817
77	Homo sapiens matrix metalloproteinase 2 (gelatinase A, 72kDa gelatinase, 72kDa type IV collagenase) (MMP2), mRNA	NM_004530	MMP2	0.0012	0.5798
78	Peptidylprolyl isomerase C (cyclophilin C), binds cyclosporin A, has peptidylprolyl isomerase as well as nuclease activity, and may inflict DNA damage during apoptosis	I_963371	I_963371	0.004	0.5778
79	Homo sapiens procollagen-proline, 2-oxoglutarate 4-dioxygenase (proline 4-hydroxylase), alpha polypeptide II (P4HA2), mRNA	NM_004199	P4HA2	0.0027	0.5762
80	Thioredoxin reductase 1, member of the pyridine nucleotide-disulfide oxidoreductase family with FAD and NADPH binding domains, has roles in maintaining redox balance, in cell growth and in apoptosis, may be involved in cancer and autoimmune disease	I_946596	TXNRD1	0.004	0.5748
81	Homo sapiens Cbp/p300-interacting transactivator, with Glu/Asp-rich carboxy-terminal domain, 2 (CITED2), mRNA	NM_006079	CITED2	0.0011	0.5733
82	Homo sapiens phosphohistidine phosphatase (PHP14), mRNA	NM_014172	PHP14	0.0018	0.5732
83	Homo sapiens selenoprotein SelM (SELM), mRNA	NM_080430	SELM	0.0032	0.5706
84	Homo sapiens methyl-CpG binding domain-containing protein MBD3 (MBD3) mRNA, complete cds	AF072247	AF072247	0.0013	0.568
85	Homo sapiens cDNA clone IMAGE:4452456, partial cds	BC018448	BC018448	0.0013	0.5662
86	Homo sapiens LIM domain only 7 (LMO7), transcript variant 1, mRNA	NM_005358	LMO7	0.0014	0.5652
87	Homo sapiens dermcidin (DCD), mRNA	NM_053283	DCD	0.001	0.5639
88	Homo sapiens mRNA; cDNA DKFZp761F169 (from clone DKFZp761F169)	AL833884	AL833884	0.0009	0.5628
89	Homo sapiens S100 calcium binding protein A4 (calcium protein, calvasculin, metastasin, murine placental homolog) (S100A4), transcript variant 1, mRNA	NM_002961	S100A4	0.0011	0.56

90	Homo sapiens chromosome 14 open reading frame 141 (C14orf141), mRNA	NM_032035	C14orf141	0.0036	0.5589
91	Protein with very strong similarity to rat MPL3, which is light chain 3, a subunit of microtubule-associated proteins 1A and 1B that may regulate the ability of those proteins to bind microtubules	I_959689	I_959689	0.0039	0.5572
92	Homo sapiens RIG-like 7-1 mRNA, complete cds	AF034208	DKK3	0.0031	0.557
93	Homo sapiens syntaxin 18 (STX18), mRNA	NM_016930	STX18	0.0019	0.5566
94	Homo sapiens lysyl oxidase (LOX), mRNA	NM_002317	LOX	0.002	0.552
95	Protein with very strong similarity to synaptic Ras GTPase activating protein (rat Syngap1), which may act in synaptic transmission and in the neuropathology of cerebral ischemia, member of the Ras GTPase-activating protein family, contains a C2 domain	I_1109613	I_1109613	0.001	0.5517
96	Homo sapiens cDNA FLJ11245 fis, clone PLACE1008629	AK002107	AK002107	0.0013	0.5516
97	Homo sapiens collagen, type IV, alpha 2 (COL4A2), mRNA	NM_001846	COL4A2	0.0025	0.5508
98	Human aldolase pseudogene mRNA, complete cds	M21191	M21191	0.0011	0.5489
99	Member of the glycosyl transferase family 2, contains three QXW (ricin B) lectin repeat domains, has moderate similarity to human GALNT1, which is a UDP-N-acetyl-alpha-D-galactosamine:polypeptide N-acetylgalactosaminyl transferase	I_1918393	I_1918393	0.0041	0.5483
100	AGENCOURT_8829785 NIH_MGC_141 Homo sapiens cDNA clone IMAGE:6386006 5, MRNA sequence	BU587941	BU587941	0.0019	0.5478
101	Homo sapiens cystatin D (CST5), mRNA	NM_001900	CST5	0.0016	0.5386
102	Homo sapiens follistatin (FST), transcript variant FST344, mRNA	NM_013409	FST	0.0033	0.5378
103	Homo sapiens protein expressed in thyroid (YF13H12), mRNA	NM_014297	ETHE1	0.0012	0.5369
104	Homo sapiens hypothetical protein FLJ14054 (FLJ14054), mRNA	NM_024563	FLJ14054	0.0012	0.5368
105	Homo sapiens integrin, beta-like 1 (with EGF-like repeat domains) (ITGBL1), mRNA	NM_004791	ITGBL1	0.0037	0.536
106	Homo sapiens ATPase, H+ transporting, lysosomal 16kDa, V0 subunit c (ATP6V0C), mRNA	NM_001694	ATP6V0C	0.0027	0.5336
107	Homo sapiens GABA(A) receptor-associated protein (GABARAP), mRNA	NM_007278	GABARAP	0.0045	0.5326
108	Homo sapiens GARS-AIRS-GART mRNA, partial cds	AF008655	GART	0.0023	0.5293
109	Homo sapiens tropomyosin 1 (alpha) (TPM1), mRNA	NM_000366	TPM1	0.0025	0.5293
110	Homo sapiens mRNA for KIAA1067 protein, partial cds	AB028990	AB028990	0.0014	0.5261
111	Homo sapiens CD81 antigen (target of antiproliferative antibody 1) (CD81), mRNA	NM_004356	CD81	0.0041	0.5261
112	Homo sapiens ferritin, light polypeptide (FTL), mRNA	NM_000146	FTL	0.0042	0.5241
113	Homo sapiens fibronectin 1 (FN1), transcript variant 1, mRNA	NM_002026	FN1	0.0015	0.524
114	Homo sapiens incomplete cDNA for a mutated allele of a myosin class I, myh-1c	AJ001381	AJ001381	0.0019	0.5206
115	Homo sapiens signal sequence receptor, delta (translocon-associated protein delta) (SSR4), mRNA	NM_006280	SSR4	0.0039	0.5199
116	Homo sapiens ras homolog gene family, member C (ARHC), mRNA	NM_175744	ARHC	0.0016	0.5166
117	Homo sapiens similar to cytosolic acyl coenzyme A thioester hydrolase (LOC344967), mRNA	XM_293652	XM_293652	0.0032	0.5154
118	Homo sapiens hypothetical protein FLJ34512 (FLJ34512), mRNA	NM_173476	FLJ34512	0.0017	0.5126
119	Forkhead box D2, a member of the HNF-3-forkhead family of predicted transcriptional regulators that may play roles in embryonic and kidney development, maintenance of cellular differentiation, and in tumorigenesis	I_930042	I_930042	0.0042	0.5098
120	Homo sapiens tumor necrosis factor receptor superfamily, member 6 (TNFRSF6), transcript variant 1, mRNA	NM_000043	TNFRSF6	0.0027	0.5063
121	Homo sapiens sodium channel, voltage-gated, type I, beta (SCN1B), mRNA	NM_001037	SCN1B	0.0039	0.5053
122	Homo sapiens ran binding protein RanBP20 mRNA, complete cds	AY026388	AY026388	0.0026	0.505
123	Homo sapiens tripartite motif-containing 22, mRNA (cDNA clone MGC:44863 IMAGE:5583800), complete cds	BC035582	BC035582	0.0024	0.5035
124	Homo sapiens brain cell membrane protein 1 (BCMP1), mRNA	NM_031442	TM4SF10	0.0044	0.5023
125	Homo sapiens U2 small nuclear ribonucleoprotein auxiliary factor, small subunit 1 (U2AF1RS1), mRNA	NM_005083	U2AF1L1	0.0028	0.5017
126	HEXA {HEXA4bpDeltaA mutation, exon 11} [human, Tay-Sachs disease patient, mRNA Partial Mutant, 78 nt]	S76980	S76980	0.0034	0.5017
127	Homo sapiens prefoldin 5 (PFDN5), transcript variant 1, mRNA	NM_002624	PFDN5	0.0043	0.5011
128	Homo sapiens FK506 binding protein 10, 65 kDa (FKBP10), mRNA	NM_021939	FKBP10	0.0028	0.4987
129	Unknown	A_32_BS10403	A_32_BS10403	0.0045	0.4961
130	Homo sapiens POU domain, class 1, transcription factor 1 (Pit1, growth hormone factor 1) (POU1F1), mRNA	NM_000306	POU1F1	0.0027	0.4955
131	Homo sapiens DKFZp564D177 protein (DKFZp564D177), mRNA	NM_015469	NIPSNAP3A	0.0039	0.4939
132	Unknown	THC1448686	THC1448686	0.0033	0.4932
133	Homo sapiens S100 calcium binding protein A13 (S100A13), mRNA	NM_005979	NM_005979	0.003	0.492
134	Homo sapiens cDNA FLJ30359 fis, clone BRACE2007760, highly similar to 40S RIBOSOMAL PROTEIN S15A	AK054921	AK054921	0.0024	0.4861
135	Homo sapiens procollagen-proline, 2-oxoglutarate 4-dioxygenase (proline 4-hydroxylase), beta polypeptide (protein disulfide isomerase; thyroid hormone binding protein p55) (P4HB), mRNA	NM_000918	P4HB	0.0029	0.4828
136	Homo sapiens desmoplakin (DSP), mRNA	NM_004415	DSP	0.0032	0.4818
137	Homo sapiens crystallin, gamma N (CRYGN), mRNA	NM_144727	CRYGN	0.0029	0.4813
138	Homo sapiens KIAA1913 protein (KIAA1913), mRNA	NM_052913	KIAA1913	0.0033	0.4813
139	Homo sapiens discoidin domain receptor family, member 2 (DDR2), mRNA	NM_006182	DDR2	0.0043	0.4775
140	Homo sapiens glutamate receptor, metabotropic 4 (GRM4), mRNA	NM_000841	GRM4	0.0042	0.4729
141	Homo sapiens cDNA: FLJ21592 fis, clone COL07036	AK025245	AK025245	0.0041	0.467
142	Homo sapiens centaurin, gamma 3 (CENTG3), mRNA	NM_031946	CENTG3	0.0033	0.4665
143	Homo sapiens ATPase, H+ transporting, lysosomal 14kDa, V1 subunit F (ATP6V1F), mRNA	NM_004231	ATP6V1F	0.0038	0.4661
144	Unknown	ENST00000332699	ENST00000332699	0.0036	0.466
145	Homo sapiens glucocorticoid modulatory element binding protein 1 (GMEB1), transcript variant 1, mRNA	NM_006582	GMEB1	0.0032	0.4633
146	Homo sapiens solute carrier family 16 (monocarboxylic acid transporters), member 3 (SLC16A3), mRNA	NM_004207	SLC16A3	0.0049	0.4628
147	Homo sapiens secreted protein, acidic, cysteine-rich (osteonectin) (SPARC), mRNA	NM_003118	SPARC	0.0042	0.4602
148	Homo sapiens caldesmon 1, transcript variant 3, mRNA (cDNA clone MGC:21352 IMAGE:4753285), complete cds	BC040354	BC040354	0.0039	0.4597
149	Homo sapiens, clone IMAGE:4791553, mRNA	BC045778	BC045778	0.0032	0.4595
150	Homo sapiens myosin, light polypeptide 5, regulatory (MYL5), mRNA	NM_002477	MYL5	0.0042	0.4592
151	Unknown	XM_295799	XM_295799	0.0039	0.459
152	Homo sapiens alveolar soft part sarcoma chromosome region, candidate 1 (ASPSCR1), mRNA	NM_024083	ASPSCR1	0.0048	0.4587
153	Unknown	A_32_BS163368	A_32_BS163368	0.0046	0.4572
154	shee129d-sheec.down Human esophageal cancer differentially expressed library Homo sapiens cDNA, MRNA sequence	BF059771	BF059771	0.0041	0.4557
155	Homo sapiens hypothetical protein BC016005 (LOC129642), mRNA	NM_138799	LOC129642	0.0048	0.4557
156	Homo sapiens dickkopf homolog 1 (Xenopus laevis) (DKK1), mRNA	NM_012242	DKK1	0.004	0.4548
157	Homo sapiens cDNA FLJ37525 fis, clone BRCAN2009156, moderately similar to Xenopus laevis mRNA for Nfrl	AK094844	AK094844	0.0041	0.4548
158	Unknown	ENST00000331598	ENST00000331598	0.0039	0.4524
159	Homo sapiens cysteine and glycine-rich protein 1 (CSRP1), mRNA	NM_004078	CSRP1	0.0037	0.4501
160	Homo sapiens hypothetical protein DKFZp586M1120, mRNA (cDNA clone MGC:34123 IMAGE:5172360), complete cds	BC040276	BC040276	0.0042	0.4496
161	Oxytocin receptor, a G protein-coupled receptor, signals through phospholipase C, functions in uterine muscle contraction, likely acts in parturition and lactation; agonist stimulation may inhibit cell proliferation in breast cancer	I_944062	OXTR	0.0037	0.4481
162	Homo sapiens protein tyrosine phosphatase-like (proline instead of catalytic arginine), member a (PTPLA), mRNA	NM_014241	PTPLA	0.0049	0.4476
163	Unknown	ENST00000331369	ENST00000331369	0.0047	0.4356
164	oe54g02.s1 NCI_CGAP_Lu5 Homo sapiens cDNA clone IMAGE:1415474 3 similar to TR:Q15157 Q15157 PMS4 MRNA ;, MRNA sequence	AA770647	AA770647	0.0049	0.4295

171 Down-regulated Significant Genes (ANOVA, p-value less than 0.005)

NN	Description	GB accession	Gene symbol	Parametric p-value	Log-fold change (^{125}IUdR/^{127}IUdR)
1	Homo sapiens keratin associated protein 19-1 (KRTAP19-1), mRNA	NM_181607	KRTAP19-1	0.0046	-0.4314
2	Unknown	XM_208135	XM_208135	0.0045	-0.4354
3	Homo sapiens heat shock 90kDa protein 1, alpha (HSPCA), mRNA	NM_005348	HSPCA	0.0046	-0.4401
4	AUTO: Very strong regional similarity to (Homo sapiens) OK_SW-cl.56: Protein with strong similarity to tubulin beta 2 (rat Tubb2), which polymerizes to form microtubules and binds alpha-tubulin (rat Tuba1) at the end of mitosis, member of the ...	I_1901891	I_1901891	0.0049	-0.4478
5	Homo sapiens nucleolin (NCL), mRNA	NM_005381	NCL	0.0038	-0.4495
6	Homo sapiens chaperonin containing TCP1, subunit 6A (zeta 1) (CCT6A), mRNA	NM_001762	CCT6A	0.0044	-0.452
7	Homo sapiens tubulin, alpha-like 2 (TUBAL2), mRNA	NM_018943	TUBA8	0.0043	-0.4554
8	Homo sapiens calcyclin binding protein mRNA, complete cds	AF057356	AF057356	0.0047	-0.459
9	Homo sapiens histone 3, H3 (HIST3H3), mRNA	NM_003493	HIST3H3	0.0041	-0.46
10	Homo sapiens cDNA FLJ13736 fis, clone PLACE3000156	AK023798	AK023798	0.0033	-0.4632
11	Homo sapiens tubulin beta-5 (TUBB-5), mRNA	NM_032525	MGC4083	0.0029	-0.465
12	Unknown	XM_301830	XM_301830	0.0041	-0.4651
13	Homo sapiens hypoxanthine phosphoribosyltransferase 1 (Lesch-Nyhan syndrome) (HPRT1), mRNA	NM_000194	HPRT1	0.0039	-0.4663
14	Homo sapiens hypothetical protein FLJ20364 (FLJ20364), mRNA	NM_017785	FLJ20364	0.004	-0.467
15	Unknown	ENST00000326269	ENST00000326269	0.0043	-0.469
16	Homo sapiens heterogeneous nuclear ribonucleoprotein U (scaffold attachment factor A) (HNRPU), transcript variant 1, mRNA	NM_031844	HNRPU	0.0027	-0.4707
17	Homo sapiens phosphoribosylaminoimidazole carboxylase, phosphoribosylaminoimidazole succinocarboxamide	NM_006452	PAICS	0.0026	-0.4757

	synthetase (PAICS), mRNA				
18	Homo sapiens cDNA FLJ14320 fis, clone PLACE3000455	AK024382	AK024382	0.0034	-0.4769
19	Homo sapiens dynein, axonemal, intermediate polypeptide 2 (DNAI2), mRNA	NM_023036	DNAI2	0.0029	-0.4771
20	Homo sapiens LSM5 homolog, U6 small nuclear RNA associated (S. cerevisiae) (LSM5), mRNA	NM_012322	LSM5	0.003	-0.4775
21	Homo sapiens tubulin, beta, 2 (TUBB2), mRNA	NM_006088	TUBB2	0.0028	-0.4807
22	Homo sapiens deoxycytidine kinase (DCK), mRNA	NM_000788	DCK	0.0023	-0.4831
23	Homo sapiens splicing factor, arginine/serine-rich 2 (SFRS2), mRNA	NM_003016	SFRS2	0.0044	-0.4862
24	AUTO: Strong similarity to (Homo sapiens) RBMX: X chromosome RNA binding motif protein, may possess X chromosome specific RNA binding activity	I_1896546	I_1896546	0.0035	-0.4874
25	Homo sapiens testis mitotic checkpoint BUB3 (BUB3) mRNA, complete cds	AF047473	AF047473	0.0022	-0.4892
26	Unknown	ENST00000328209	ENST00000328209	0.0021	-0.4893
27	Unknown	ENST00000299806	ENST00000299806	0.0027	-0.4937
28	Homo sapiens solute carrier family 25 (mitochondrial carrier; adenine nucleotide translocator), member 5 (SLC25A5), mRNA	NM_001152	SLC25A5	0.0028	-0.4945
29	Human intercrine-alpha (hIRH) mRNA, complete cds	U19495	U19495	0.0025	-0.4955
30	Homo sapiens protein phosphatase 1, catalytic subunit, gamma isoform (PPP1CC), mRNA	NM_002710	PPP1CC	0.0022	-0.4964
31	Homo sapiens tyrosine 3-monooxygenase/tryptophan 5-monooxygenase activation protein, theta polypeptide (YWHAQ), mRNA	NM_006826	YWHAQ	0.0018	-0.5021
32	Homo sapiens cyclin A2 (CCNA2), mRNA	NM_001237	CCNA2	0.0028	-0.5028
33	Homo sapiens cell division cycle 2-like 2 (CDC2L2), transcript variant 6, mRNA	NM_033532	CDC2L2	0.002	-0.5058
34	Homo sapiens ADP-ribosylation factor-like 6 interacting protein (ARL6IP), mRNA	NM_015161	ARL6IP	0.0039	-0.5063
35	Unknown	ENST00000311040	ENST00000311040	0.0033	-0.5066
36	Unknown	ENST00000305471	ENST00000305471	0.0017	-0.5084
37	Homo sapiens histone acetyltransferase 1 (HAT1), mRNA	NM_003642	HAT1	0.0023	-0.5101
38	Homo sapiens tyrosine 3-monooxygenase/tryptophan 5-monooxygenase activation protein, eta polypeptide (YWHAH), mRNA	NM_003405	YWHAH	0.0018	-0.5126
39	Homo sapiens catenin (cadherin-associated protein), alpha-like 1 (CTNNAL1), mRNA	NM_003798	CTNNAL1	0.0017	-0.5221
40	Homo sapiens DEAH (Asp-Glu-Ala-His) box polypeptide 9 (DHX9), transcript variant 1, mRNA	NM_001357	DHX9	0.0017	-0.525
41	AUTO: Protein of unknown function	I_3216774	I_3216774	0.0016	-0.5253
42	Homo sapiens heterogeneous nuclear ribonucleoprotein A2/B1 (HNRPA2B1), transcript variant A2, mRNA	NM_002137	HNRPA2B1	0.0035	-0.5254
43	AUTO: Very strong regional similarity to (Mus musculus) MGC37309: Protein of unknown function, has high similarity to a region of heterogeneous nuclear ribonucleoprotein A1 (human HNRPA1), which is an RNA-binding protein involved in mRNA splicing an ...	I_3232524	I_3232524	0.0025	-0.5267
44	Homo sapiens clone 2 CC chemokine receptor 3 (CCR3) mRNA, partial cds	AF262300	CCR3	0.0012	-0.5327
45	Unknown	ENST00000326507	ENST00000326507	0.0014	-0.5336
46	Homo sapiens H3 histone, family 3B (H3.3B) (H3F3B), mRNA	NM_005324	H3F3B	0.0013	-0.5354
47	Unknown	ENST00000321800	ENST00000321800	0.003	-0.5356
48	Homo sapiens heterogeneous nuclear ribonucleoprotein A1 (HNRPA1), transcript variant 2, mRNA	NM_031157	HNRPA1	0.0018	-0.5392
49	AUTO: Strong regional similarity to (Homo sapiens) TEBP: Inactive progesterone receptor (23kD), cochaperone with prostaglandin E synthase activity that is a component of the unstimulated progesterone receptor complex and that enhances receptor compl ...	I_3300899	I_3300899	0.0013	-0.5405
50	Unknown	ENST00000313760	ENST00000313760	0.0021	-0.5414
51	Homo sapiens tubulin alpha 6 (TUBA6), mRNA	NM_032704	TUBA6	0.0011	-0.5438
52	Unknown	ENST00000331111	ENST00000331111	0.0013	-0.5461
53	Homo sapiens heterogeneous nuclear ribonucleoprotein F (HNRPF), mRNA	NM_004966	HNRPF	0.0011	-0.5477
54	AUTO: Protein of unknown function	I_3545016	I_3545016	0.0017	-0.5548
55	AUTO: Protein of unknown function	I_1842252	I_1842252	0.0014	-0.5579
56	Homo sapiens kinesin family member 11 (KIF11), mRNA	NM_004523	KIF11	0.0038	-0.558
57	Homo sapiens Smith-Magenis syndrome chromosome region, candidate 5 (SMCR5), mRNA	NM_144774	SMCR5	0.0027	-0.56
58	Homo sapiens ADP-ribosyltransferase (NAD+; poly (ADP-ribose) polymerase) (ADPRT), mRNA	NM_001618	ADPRT	0.0018	-0.5603
59	Homo sapiens cell division cycle 2, G1 to S and G2 to M (CDC2), transcript variant 1, mRNA	NM_001786	CDC2	0.0036	-0.5631
60	Homo sapiens retinoblastoma binding protein 7 (RBBP7), mRNA	NM_002893	RBBP7	0.001	-0.5634
61	Homo sapiens cyclin-dependent kinase inhibitor 3 (CDK2-associated dual specificity phosphatase) (CDKN3), mRNA	NM_005192	CDKN3	0.0043	-0.5661
62	Homo sapiens likely ortholog of mouse tubulin alpha 4 (FLJ13940), mRNA	NM_025019	TUBA4	0.001	-0.5663
63	Human ADP/ATP carrier protein mRNA, complete cds	J02683	J02683	0.0009	-0.5695
64	Unknown	ENST00000318026	ENST00000318026	0.001	-0.5756
65	ac68e12.s1 Stratagene fetal retina 937202 Homo sapiens cDNA clone IMAGE:867790 3 similar to gb:X12671_rna1 HETEROGENEOUS NUCLEAR RIBONUCLEOPROTEIN A1 (HUMAN);, MRNA sequence	AA780756	AA780756	0.0008	-0.5768
66	Homo sapiens replication protein A1, 70kDa (RPA1), mRNA	NM_002945	RPA1	0.0007	-0.5851
67	Homo sapiens cDNA FLJ25344 fis, clone TST01087	AK058073	AK058073	0.0011	-0.5893
68	Homo sapiens similar to unactive progesterone receptor, 23 kD; likely ortholog of mouse telomerase binding protein, p23 (LOC284672), mRNA	XM_208234	XM_208234	0.0008	-0.59
69	Homo sapiens chromobox homolog 1 (HP1 beta homolog Drosophila) (CBX1), mRNA	NM_006807	CBX1	0.0006	-0.6022
70	Homo sapiens ribonuclease P1 (RNASEP1), mRNA	NM_006638	RNASEP1	0.0012	-0.6036
71	Homo sapiens cDNA clone IMAGE:4448513, partial cds	BC046178	BC046178	0.001	-0.6037
72	Homo sapiens hypothetical protein LOC339768, mRNA (cDNA clone IMAGE:5552130), partial cds	BC042051	BC042051	0.0011	-0.6076
73	Homo sapiens splicing factor, arginine/serine-rich 3 (SFRS3), mRNA	NM_003017	SFRS3	0.0005	-0.6077
74	Homo sapiens full length insert cDNA clone YP08F12	AF085874	AF085874	0.0005	-0.6091
75	Human deoxyuridine nucleotidohydrolase mRNA, complete cds	U31930	DUT	0.0006	-0.6136
76	Human fibroblast growth factor-5 (FGF-5) mRNA, complete cds	M37825	M37825	0.0009	-0.6137
77	Protein with high similarity to human HNRPA1, which is a heterogeneous nuclear ribonucleoprotein that may function as an RNA carrier during export from the nucleus to the cytoplasm, contains an RNA recognition motif (RRM, RBD, or RNP) domain	I_932099	I_932099	0.0005	-0.6163
78	Homo sapiens PC4 and SFRS1 interacting protein 2 (PSIP2), mRNA	NM_033222	PSIP2	0.0005	-0.6264
79	Homo sapiens epithelial cell transforming sequence 2 oncogene (ECT2), mRNA	NM_018098	ECT2	0.0038	-0.633
80	Human translation initiation factor 5 (eIF5) mRNA, complete cds	U49436	U49436	0.0003	-0.6382
81	Homo sapiens ribonucleotide reductase M1 polypeptide (RRM1), mRNA	NM_001033	RRM1	0.0006	-0.6427
82	Homo sapiens PP3731 mRNA, complete cds	AF258562	AF258562	0.0031	-0.6436
83	Homo sapiens SMC4 structural maintenance of chromosomes 4-like 1 (yeast) (SMC4L1), mRNA	NM_005496	SMC4L1	0.0009	-0.647
84	Homo sapiens lamin B2 (LMNB2), mRNA	NM_032737	LMNB2	0.0004	-0.6476
85	Unknown	XM_302632	XM_302632	0.0005	-0.6529
86	AUTO: Strong regional similarity to (Homo sapiens) HNRPA1: Heterogeneous nuclear ribonucleoprotein A1, an RNA-binding protein involved in mRNA splicing and translational control, undergoes nucleocytoplasmic shuttling possibly for mRNA export and ...	I_1877093	I_1877093	0.0003	-0.656
87	Homo sapiens heterogeneous nuclear ribonucleoprotein H1 (H) (HNRPH1), mRNA	NM_005520	HNRPH1	0.0003	-0.6579
88	Homo sapiens prothymosin a14 (LOC51685), mRNA	NM_016171	NM_016171	0.0004	-0.6601
89	Unknown	ENST00000320402	ENST00000320402	0.0006	-0.6621
90	Homo sapiens chromosome 10 open reading frame 3 (C10orf3), mRNA	NM_018131	C10orf3	0.0006	-0.6635
91	Homo sapiens chemokine (C-X-C motif) ligand 12 (stromal cell-derived factor 1) (CXCL12), mRNA	NM_000609	CXCL12	0.0006	-0.6669
92	Homo sapiens histone H2A.F/Z variant (H2AV), transcript variant 1, mRNA	NM_012412	H2AV	0.0003	-0.6701
93	AUTO: Protein of unknown function	I_1871320	I_1871320	0.0046	-0.6772
94	Homo sapiens heterogeneous nuclear ribonucleoprotein D (AU-rich element RNA binding protein 1, 37kDa) (HNRPD), transcript variant 1, mRNA	NM_031370	HNRPD	0.0003	-0.68
95	Prothymosin alpha, a nuclear protein associated with various components of cell proliferation and differentiation, may act in and serve as a marker for the development and metastasis of various cancers, may be a marker for poor prognosis in breast c ...	I_958468	I_958468	0.0008	-0.6829
96	Homo sapiens ubiquitin specific protease 1 (USP1), mRNA	NM_003368	USP1	0.0002	-0.6862
97	Unknown	ENST00000330305	ENST00000330305	0.0002	-0.6896
98	Homo sapiens CSE1 chromosome segregation 1-like (yeast) (CSE1L), transcript variant 1, mRNA	NM_001316	CSE1L	0.0002	-0.7098
99	Homo sapiens proliferating cell nuclear antigen (PCNA), transcript variant 1, mRNA	NM_002592	PCNA	0.0001	-0.7356
100	Homo sapiens RAD21 homolog (S. pombe) (RAD21), mRNA	NM_006265	RAD21	0.0002	-0.7526
101	Homo sapiens cDNA FLJ31079 fis, clone HSYRA2001595	AK055641	AK055641	0.0001	-0.7533
102	Homo sapiens acidic (leucine-rich) nuclear phosphoprotein 32 family, member E (ANP32E), mRNA	NM_030920	ANP32E	0.0001	-0.7547
103	Unknown	THC1444500	THC1444500	0.0001	-0.7579
104	Homo sapiens cytoskeleton associated protein 2 (CKAP2), mRNA	NM_018204	CKAP2	0.0001	-0.7662
105	Unknown	ENST00000316482	ENST00000316482	0.0003	-0.7689
106	Homo sapiens anillin, actin binding protein (scraps homolog, Drosophila) (ANLN), mRNA	NM_018685	ANLN	0.0001	-0.7719
107	Homo sapiens heterogeneous nuclear ribonucleoprotein R (HNRPR), mRNA	NM_005826	HNRPR	0.0002	-0.7729
108	AUTO: Very strong regional similarity to (Homo sapiens) HNRPA1: Heterogeneous nuclear ribonucleoprotein A1, an RNA-binding protein involved in mRNA splicing and translational control, undergoes nucleocytoplasmic shuttling possibly for	I_2021259	I_2021259	0.0001	-0.787

	mRNA export an ...				
109	Homo sapiens MAD2 mitotic arrest deficient-like 1 (yeast) (MAD2L1), mRNA	NM_002358	MAD2L1	0.0001	-0.7917
110	Homo sapiens high-mobility group box 1 (HMGB1), mRNA	NM_002128	HMGB1	0.0001	-0.7941
111	Homo sapiens cyclin-dependent kinase inhibitor 2C (p18, inhibits CDK4) (CDKN2C), transcript variant 1, mRNA	NM_001262	CDKN2C	0.0007	-0.7996
112	Homo sapiens ADP-ribosylation factor-like 6 interacting protein, mRNA (cDNA clone MGC:5360 IMAGE:3048642), complete cds	BC010281	ARL6IP	0.0002	-0.8019
113	Unknown	ENST00000329199	ENST00000329199	0.0001	-0.8112
114	Homo sapiens CDC28 protein kinase regulatory subunit 1B (CKS1B), mRNA	NM_001826	CKS1B	0.0003	-0.8241
115	Homo sapiens ZW10 interactor (ZWINT), transcript variant 2, mRNA	NM_032997	ZWINT	0.0003	-0.8403
116	AUTO: Protein of unknown function	I_1938489	I_1938489	0.0001	-0.8484
117	603389224F1 NIH_MGC_87 Homo sapiens cDNA clone IMAGE:5398493 5, MRNA sequence	BI858892	BI858892	<0.0001	-0.8546
118	Unknown	THC1595926	THC1595926	0.0001	-0.862
119	Unknown	ENST00000322901	ENST00000322901	0.0001	-0.8688
120	AUTO: Very strong similarity to (Homo sapiens) HMGN2: High-mobility group (nonhistone chromosomal) protein 17, member of the HMGN family, reduces chromatin compaction by binding to nucleosomes, enhances transcription and possibly DNA replication; ...	I_1916733	I_1916733	<0.0001	-0.8705
121	DEK oncogene, DNA- and RNA-binding protein, alters chromatin topology and interacts with splicing factors, autoantigens are linked to juvenile rheumatoid arthritis and systemic lupus erythematosus; gene fusion with CAN is seen in acute myeloid leukemia	I_957136	I_957136	<0.0001	-0.8727
122	Unknown	ENST00000327364	ENST00000327364	0.0001	-0.8776
123	Unknown	NM_021024	NM_021024	<0.0001	-0.879
124	PROTHYMOSIN A14. [Source:RefSeq;Acc:NM_016171]	ENST00000324717	ENST00000324717	0.0001	-0.881
125	Unknown	ENST00000326004	ENST00000326004	0.0001	-0.8861
126	CYCLIN-DEPENDENT KINASES REGULATORY SUBUNIT 1 (CKS-1) (SID1334) (PNAS-16 / PNAS-143). [Source:SWISSPROT;Acc:P33551]	ENST00000325544	ENST00000325544	<0.0001	-0.8906
127	Homo sapiens cyclin B2 (CCNB2), mRNA	NM_004701	CCNB2	0.0001	-0.9046
128	Unknown	ENST00000314726	ENST00000314726	<0.0001	-0.9142
129	Unknown	ENST00000331466	ENST00000331466	<0.0001	-0.9143
130	Homo sapiens high-mobility group box 1, mRNA (cDNA clone MGC:5223 IMAGE:2901382), complete cds	BC003378	HMGB1	0.0001	-0.9199
131	Unknown	ENST00000330913	ENST00000330913	<0.0001	-0.9223
132	AUTO: Very strong similarity to (Homo sapiens) HMGN2: High-mobility group (nonhistone chromosomal) protein 17, member of the HMGN family, reduces chromatin compaction by binding to nucleosomes, enhances transcription and possibly DNA replication; ...	I_2001380	I_2001380	<0.0001	-0.932
133	Homo sapiens ubiquitin UBF-fl (UBF-fl), mRNA	NM_032828	ZF6	<0.0001	-0.9353
134	Unknown	XM_301836	XM_301836	<0.0001	-0.9481
135	High-mobility group (nonhistone chromosomal) protein 17, member of the HMGN family, reduces chromatin compaction by binding to nucleosomes, enhances transcription and possibly DNA replication; corresponding gene may be mutated in various neoplasms	I_1221897	I_1221897	<0.0001	-0.9593
136	Homo sapiens kinesin family member 23 (KIF23), transcript variant 1, mRNA	NM_138555	KIF23	0.0001	-0.9603
137	Unknown	THC1529347	THC1529347	<0.0001	-0.9817
138	Homo sapiens histone 1, H4c (HIST1H4C), mRNA	NM_003542	HIST1H4C	0.0003	-0.9819
139	DJ579F20.1 (HIGH-MOBILITY GROUP (NONHISTONE CHROMOSOMAL) PROTEIN 1-LIKE 1). [Source:SPTREMBL;Acc:Q9NQJ4]	ENST00000243919	ENST00000243919	0.0001	-0.9823
140	Unknown	XM_291821	XM_291821	<0.0001	-0.9886
141	Homo sapiens hypothetical protein FLJ39426 (FLJ39426), mRNA	NM_173609	FLJ39426	<0.0001	-0.9909
142	Unknown	ENST00000328536	ENST00000328536	<0.0001	-1.0034
143	Unknown	THC1595925	THC1595925	<0.0001	-1.0371
144	Unknown	ENST00000328021	ENST00000328021	<0.0001	-1.0443
145	Homo sapiens ribonucleotide reductase M2 polypeptide, mRNA (cDNA clone MGC:29692 IMAGE:4858919), complete cds	BC030154	RRM2	0.0001	-1.0649
146	Unknown	ENST00000261249	ENST00000261249	<0.0001	-1.07
147	Unknown	ENST00000331549	ENST00000331549	<0.0001	-1.1043
148	Homo sapiens protein regulator of cytokinesis 1 (PRC1), mRNA	NM_003981	PRC1	<0.0001	-1.1128
149	Homo sapiens high-mobility group nucleosomal binding domain 2, mRNA (cDNA clone MGC:29711 IMAGE:5021546), complete cds	BC032140	BC032140	<0.0001	-1.1157
150	AUTO: Strong similarity to (Homo sapiens) CKS1B: CDC28 protein kinase 1, binds and regulates CDK2-cyclin A complexes, similar to S. pombe p13suc1, required for SCF dependent degradation of p27	I_3537291	I_3537291	<0.0001	-1.125
151	Homo sapiens cDNA FLJ40937 fis, clone UTERU2007499	AK098256	AK098256	<0.0001	-1.13
152	AUTO: Protein of unknown function	I_1969309	I_1969309	<0.0001	-1.1533
153	Homo sapiens pituitary tumor-transforming 2 (PTTG2), mRNA	NM_006607	PTTG2	<0.0001	-1.1539
154	AUTO: Protein of unknown function	I_1904481	I_1904481	<0.0001	-1.1636
155	Homo sapiens karyopherin alpha 2 (RAG cohort 1, importin alpha 1) (KPNA2), mRNA	NM_002266	KPNA2	0.0001	-1.1686
156	Homo sapiens prothymosin, alpha (gene sequence 28) (PTMA), mRNA	NM_002823	PTMA	<0.0001	-1.1705
157	Homo sapiens thymidylate synthetase (TYMS), mRNA	NM_001071	TYMS	<0.0001	-1.1818
158	Homo sapiens Rac GTPase activating protein 1 (RACGAP1), mRNA	NM_013277	RACGAP1	<0.0001	-1.2173
159	Homo sapiens nuclear antigen Sp100 (SP100), mRNA	NM_003113	SP100	<0.0001	-1.2308
160	Homo sapiens pituitary tumor-transforming 1 (PTTG1), mRNA	NM_004219	PTTG1	<0.0001	-1.234
161	Homo sapiens ubiquitin-conjugating enzyme E2C (UBE2C), transcript variant 6, mRNA	NM_181803	UBE2C	<0.0001	-1.2691
162	Homo sapiens CDC28 protein kinase regulatory subunit 2 (CKS2), mRNA	NM_001827	CKS2	<0.0001	-1.2955
163	Unknown	ENST00000325202	ENST00000325202	<0.0001	-1.3142
164	Homo sapiens H2A histone family, member Z (H2AFZ), mRNA	NM_002106	H2AFZ	<0.0001	-1.3275
165	Homo sapiens H2A histone family, member X (H2AFX), mRNA	NM_002105	H2AFX	<0.0001	-1.3354
166	Homo sapiens cyclin B1 (CCNB1), mRNA	NM_031966	CCNB1	<0.0001	-1.3356
167	Homo sapiens KIAA0101 gene product (KIAA0101), mRNA	NM_014736	KIAA0101	<0.0001	-1.3719
168	Unknown	XM_302460	XM_302460	<0.0001	-1.372
169	Homo sapiens ubiquitin-like, containing PHD and RING finger domains, 1 (UHRF1), mRNA	NM_013282	UHRF1	<0.0001	-1.5051
170	Homo sapiens stathmin 1/oncoprotein 18 (STMN1), mRNA	NM_005563	STMN1	<0.0001	-1.6775
171	Homo sapiens high-mobility group box 2 (HMGB2), mRNA	NM_002129	HMGB2	<0.0001	-1.731
