# Supplementary material for: Effects of DNA-targeted ionizing radiation produced by 5-[125I]iodo-2'-deoxyuridine on global gene expression in primary human cells
Source: BMC Genomics. 2007 Jun 26;8:192. doi: 10.1186/1471-2164-8-192 (PMC1934370; doi:10.1186/1471-2164-8-192)
Supplement: Additional file 2 — 125IUdR – responsive set of genes in GM05388 cell line (3.7 kBq/ml) [file 1471-2164-8-192-S2.pdf]

Supplementary table 2. ^{125}IUdR - responsive set of genes in GM05388 cell line (3.7 kBq/ml)

17 Up-regulated Significant Genes (ANOVA, p-value less than 0.005)

NN	Description	GB accession	Gene symbol	Parametric p-value	Log-fold change (^{125}IUdR/^{127}IUdR)
1	Homo sapiens cyclin-dependent kinase inhibitor 1A (p21, Cip1) (CDKN1A), transcript variant 1, mRNA	NM_000389	CDKN1A	<0.0001	1.7513
2	Homo sapiens fibroblast growth factor 2 (basic) (FGF2), mRNA	NM_002006	FGF2	0.0001	0.902
3	Homo sapiens dickkopf homolog 1 (Xenopus laevis) (DKK1), mRNA	NM_012242	DKK1	0.0002	0.8511
4	Homo sapiens tripartite motif-containing 22, mRNA (cDNA clone MGC:44863 IMAGE:5583800), complete cds	BC035582	BC035582	0.0031	0.6722
5	Homo sapiens p53-induced protein PIGPC1 (PIGPC1), mRNA	NM_022121	PERP	0.0012	0.6611
6	Homo sapiens four and a half LIM domains 2 (FHL2), mRNA	NM_001450	FHL2	0.0006	0.6485
7	Homo sapiens sparc/osteonectin, cwcv and kazal-like domains proteoglycan (testican) (SPOCK), mRNA	NM_004598	SPOCK	0.0008	0.6378
8	Homo sapiens ras homolog gene family, member E (ARHE), mRNA	NM_005168	ARHE	0.0019	0.5527
9	Homo sapiens growth arrest and DNA-damage-inducible, alpha (GADD45A), mRNA	NM_001924	GADD45A	0.0033	0.5392
10	Plasminogen activator inhibitor 1, a member of the serpin family of serine proteases and inhibitors, plays a role in regulating blood coagulation by inhibiting fibrinolysis, contributes to tumor progression and is a risk factor for cardiovascular ...	I_929648	I_929648	0.0037	0.5367
11	insulin-like growth factor binding protein 3 {3 region} [human, tuberous sclerosis cells, mRNA Partial, 704 nt]	S56205	S56205	0.0041	0.5353
12	Unknown	XM_165930	XM_165930	0.0023	0.5309
13	Homo sapiens cDNA FLJ38456 fis, clone FEBRA2019867	AK095775	AK095775	0.0031	0.5251
14	Homo sapiens ras homolog gene family, member E (ARHE), mRNA	NM_005168	ARHE	0.0032	0.4899
15	Homo sapiens mRNA for TGF-betaIIR alpha, complete cds	D50683	D50683	0.004	0.4823
16	Homo sapiens GARS-AIRS-GART mRNA, partial cds	AF008655	GART	0.0049	0.4802
17	Homo sapiens amphoterin induced gene 2 (AMIGO2), mRNA	NM_181847	AMIGO2	0.0043	0.4724

33 Down-regulated Significant Genes (ANOVA, p-value less than 0.005)

NN	Description	GB accession	Gene symbol	Parametric p-value	Log-fold change (^{125}IUdR/^{127}IUdR)
1	Unknown	ENST00000319053	ENST00000319053	0.0048	-0.4623
2	CYCLIN-DEPENDENT KINASES REGULATORY SUBUNIT 1 (CKS-1) (SID1334) (PNAS-16 / PNAS-143). [Source:SWISSPROT;Acc:P33551]	ENST00000325544	ENST00000325544	0.0045	-0.473
3	Homo sapiens karyopherin (importin) beta 1, mRNA (cDNA clone MGC:2155 IMAGE:3162957), complete cds	BC036703	KPNB1	0.005	-0.4769
4	Homo sapiens ADP-ribosylation factor-like 6 interacting protein, mRNA (cDNA clone MGC:5360 IMAGE:3048642), complete cds	BC010281	ARL6IP	0.0041	-0.4795
5	Homo sapiens Humanin (HN1) mRNA, complete cds	AY029066	AY029066	0.0037	-0.4801
6	Homo sapiens prothymosin a14 (LOC51685), mRNA	NM_016171	NM_016171	0.0035	-0.4995
7	Homo sapiens tubulin alpha 6, mRNA (cDNA clone MGC:45598 IMAGE:3926854), complete cds	BC033064	BC033064	0.0049	-0.5046
8	Homo sapiens tubulin, alpha 3 (TUBA3), mRNA	NM_006009	TUBA3	0.0034	-0.5072
9	Homo sapiens alpha tubulin-like (MGC16703), mRNA	NM_145042	MGC16703	0.0043	-0.5188
10	Homo sapiens DEAH (Asp-Glu-Ala-His) box polypeptide 9 (DHX9), transcript variant 1, mRNA	NM_001357	DHX9	0.0029	-0.5235
11	Homo sapiens mRNA; cDNA DKFZp434I225 (from clone DKFZp434I225); partial cds	AL117637	AL117637	0.004	-0.5582
12	Unknown	ENST00000328536	ENST00000328536	0.0035	-0.5761
13	Human fetal troponin T 1 mRNA, partial cds	U14641	TNNT3	0.002	-0.5997
14	PROTHYMOSIN A14. [Source:RefSeq;Acc:NM_016171]	ENST00000324717	ENST00000324717	0.0016	-0.6055
15	Homo sapiens high-mobility group box 1, mRNA (cDNA clone MGC:5223 IMAGE:2901382), complete cds	BC003378	HMGB1	0.0035	-0.6116
16	AUTO: Protein of unknown function	I_1938489	I_1938489	0.0044	-0.6134
17	AUTO: Protein of unknown function	I_1871320	I_1871320	0.0034	-0.6466
18	Unknown	THC1595926	THC1595926	0.0024	-0.6557
19	Unknown	THC1595925	THC1595925	0.0018	-0.6575
20	Unknown	ENST00000328021	ENST00000328021	0.0031	-0.6636
21	Unknown	ENST00000330913	ENST00000330913	0.003	-0.6686
22	Homo sapiens CDC28 protein kinase regulatory subunit 1B (CKS1B), mRNA	NM_001826	CKS1B	0.003	-0.7036
23	Homo sapiens cDNA FLJ40937 fis, clone UTERU2007499	AK098256	AK098256	0.0043	-0.7126
24	Unknown	ENST00000326004	ENST00000326004	0.0038	-0.7326
25	Homo sapiens karyopherin alpha 2 (RAG cohort 1, importin alpha 1) (KPNA2), mRNA	NM_002266	KPNA2	0.004	-0.7331
26	Homo sapiens ubiquitin-like, containing PHD and RING finger domains, 1 (UHRF1), mRNA	NM_013282	UHRF1	0.0032	-0.7378
27	Homo sapiens H2A histone family, member Z (H2AFZ), mRNA	NM_002106	H2AFZ	0.0027	-0.7573
28	Unknown	XM_301836	XM_301836	0.0037	-0.7764
29	Homo sapiens H2A histone family, member X (H2AFX), mRNA	NM_002105	H2AFX	0.0039	-0.7915
30	Homo sapiens stathmin 1/oncoprotein 18 (STMN1), mRNA	NM_005563	STMN1	0.0047	-0.7925
31	AUTO: Strong similarity to (Homo sapiens) CKS1B: CDC28 protein kinase 1, binds and regulates CDK2-cyclin A complexes, similar to S. pombe p13suc1, required for SCF dependent degradation of p27	I_3537291	I_3537291	0.0024	-0.7974
32	Homo sapiens histone 1, H4c (HIST1H4C), mRNA	NM_003542	HIST1H4C	0.001	-0.9217
33	Homo sapiens high-mobility group box 2 (HMGB2), mRNA	NM_002129	HMGB2	0.0029	-0.9708
