# Supplementary material for: Effects of DNA-targeted ionizing radiation produced by 5-[125I]iodo-2'-deoxyuridine on global gene expression in primary human cells
Source: BMC Genomics. 2007 Jun 26;8:192. doi: 10.1186/1471-2164-8-192 (PMC1934370; doi:10.1186/1471-2164-8-192)
Supplement: Additional file 3 — 125IUdR – responsive set of genes in NHFK cells [file 1471-2164-8-192-S3.pdf]

Supplementary table 3. ^{125}IUdR - responsive set of genes in normal human keratinocytes (NHFK)

16 Up-regulated Significant Genes (ANOVA, p-value less than 0.005)

NN	Description	GB accession	Gene symbol	Parametric p-value	Log-fold change (^{125}IUdR/^{127}IUdR)
1	Homo sapiens seven transmembrane protein TM7SF3 (TM7SF3), mRNA	NM_016551	TM7SF3	0.0034	0.7627
2	Homo sapiens lectin, galactoside-binding, soluble, 7 (galectin 7) (LGALS7), mRNA	NM_002307	LGALS7	0.0049	0.7566
3	Human parathyroid hormone-like peptide mRNA, 3 end	M31157	M31157	0.0019	0.6552
4	Homo sapiens tripartite motif-containing 22, mRNA (cDNA clone MGC:44863 IMAGE:5583800), complete cds	BC035582	BC035582	0.0008	0.6267
5	Homo sapiens mRNA; cDNA DKFZp586O1224 (from clone DKFZp586O1224)	AL110170	AL110170	0.0031	0.6161
6	Homo sapiens parathyroid hormone-like hormone (PTHLH), mRNA	NM_002820	PTHLH	0.0026	0.5702
7	Homo sapiens emilin and multimerin-domain containing protein 1 (EMU1), mRNA	NM_133455	EMU1	0.0034	0.5428
8	Homo sapiens SH3 domain binding glutamic acid-rich protein like, mRNA (cDNA clone MGC:23745 IMAGE:4105289), complete cds	BC016709	BC016709	0.0025	0.5332
9	Homo sapiens plasmalemma vesicle associated protein (PLVAP), mRNA	NM_031310	PLVAP	0.0028	0.5293
10	Homo sapiens mRNA for hypothetical protein (C10ORF5B gene)	AJ535621	AJ535621	0.0019	0.5144
11	Homo sapiens glycogen synthase kinase 3 beta (GSK3B), mRNA	NM_002093	GSK3B	0.0033	0.4972
12	Homo sapiens ovarian cancer related protein OVN9-3 (OVN9-3) mRNA, complete cds	AF257098	AF257098	0.0047	0.4809
13	Homo sapiens LIV-1 protein, estrogen regulated (LIV-1), mRNA	NM_012319	SLC39A6	0.0035	0.4793
14	Homo sapiens cDNA FLJ39315 fis, A-OCBBF2013926	AK096634	AK096634	0.0041	0.4664
15	Unknown	THC1440941	THC1440941	0.0039	0.4487
16	Homo sapiens lysophospholipase isoform mRNA, complete cds	AF077199	LYPLA1	0.0044	0.4405

11 Down-regulated Significant Genes (ANOVA, p-value less than 0.005)

NN	Description	GB accession	Gene symbol	Parametric p-value	Log-fold change (^{125}IUdR/^{127}IUdR)
1	Unknown	ENST00000329233	ENST00000329233	0.005	-0.4391
2	Homo sapiens heme-regulated initiation factor 2-alpha kinase (HRI), mRNA	NM_014413	HRI	0.0037	-0.4505
3	Homo sapiens hypothetical protein FLJ31795 (FLJ31795), mRNA	NM_144609	FLJ31795	0.0033	-0.4853
4	Homo sapiens lymphocyte antigen 6 complex, locus D (E48), mRNA	NM_003695	E48	0.0017	-0.5152
5	Homo sapiens deoxycytidine kinase (DCK), mRNA	NM_000788	DCK	0.0025	-0.528
6	Homo sapiens MCM3 minichromosome maintenance deficient 3 (S. cerevisiae) (MCM3), mRNA	NM_002388	MCM3	0.0023	-0.5528
7	GTP binding protein 2, a member of the GP-1 family of GTPases, contains a GTP-binding motif, and may function in signal transduction, expression is stimulated by interferon-gamma treatment	I_966849	I_966849	0.0007	-0.5938
8	Unknown	THC1483599	THC1483599	0.0016	-0.6315
9	Homo sapiens KIAA0101 gene product (KIAA0101), mRNA	NM_014736	KIAA0101	0.0012	-0.638
10	Homo sapiens F-box only protein 27 (FBXO27), mRNA	NM_178820	FBXO27	0.0007	-0.6677
11	Homo sapiens cDNA FLJ39068 fis, clone NT2RP7015080	AK096387	AK096387	0.0003	-0.7037
