# Supplementary material for: Effects of DNA-targeted ionizing radiation produced by 5-[125I]iodo-2'-deoxyuridine on global gene expression in primary human cells
Source: BMC Genomics. 2007 Jun 26;8:192. doi: 10.1186/1471-2164-8-192 (PMC1934370; doi:10.1186/1471-2164-8-192)
Supplement: Additional file 4 — 125IUdR – responsive set of genes in GM05388 cell line (18.5 kBq/ml) [file 1471-2164-8-192-S4.pdf]

Supplementary table 4. ^{125}IUdR - responsive set of genes in GM05388 cell line (18.5 kBq/ml)

69 Up-regulated Significant Genes (ANOVA, p-value less than 0.005)

NN	Description	GB accession	Gene symbol	Parametric p-value	Log-fold change (^{125}IUdR/^{127}IUdR)
1	Homo sapiens cyclin-dependent kinase inhibitor 1A (p21, Cip1) (CDKN1A), transcript variant 1, mRNA	NM_000389	CDKN1A	0.0001	1.6509
2	Homo sapiens cDNA FLJ38671 fis, clone HSYRA2000332, highly similar to Human elastin gene	AK095990	AK095990	0.0019	1.0441
3	Homo sapiens growth arrest and DNA-damage-inducible, alpha (GADD45A), mRNA	NM_001924	GADD45A	0.0004	0.9498
4	Unknown	THC1460216	THC1460216	0.0018	0.932
5	Homo sapiens prostaglandin-endoperoxide synthase 2 (prostaglandin G/H synthase and cyclooxygenase), mRNA (cDNA clone MGC:9576 IMAGE:3880850), complete cds	BC013734	BC013734	0.0001	0.9271
6	Homo sapiens cDNA FLJ38569 fis, clone HCHON2006459	AK095888	AK095888	0.0013	0.9023
7	Homo sapiens dickkopf homolog 1 (Xenopus laevis) (DKK1), mRNA	NM_012242	DKK1	0.0039	0.8901
8	Homo sapiens stomatin (STOM), mRNA	NM_004099	STOM	0.0004	0.8496
9	Homo sapiens hypothetical protein MGC35578 (MGC35578), mRNA	NM_153337	SLIC1	0.0002	0.8155
10	Homo sapiens cDNA FLJ25802 fis, clone TST07145	AK098668	AK098668	0.0019	0.8101
11	Homo sapiens serum-inducible kinase (SNK), mRNA	NM_006622	SNK	0.0015	0.8024
12	Homo sapiens cDNA FLJ11245 fis, clone PLACE1008629	AK002107	AK002107	0.0008	0.799
13	Homo sapiens hypothetical protein MGC10120 (MGC10120), mRNA	NM_173809	MGC10120	0.0003	0.77
14	Homo sapiens four and a half LIM domains 2 (FHL2), mRNA	NM_001450	FHL2	0.0003	0.7303
15	Homo sapiens tripartite motif-containing 22, mRNA (cDNA clone MGC:44863 IMAGE:5583800), complete cds	BC035582	BC035582	0.0029	0.7279
16	Homo sapiens epithelial membrane protein 1 (EMP1), mRNA	NM_001423	EMP1	0.0003	0.7199
17	Homo sapiens tissue inhibitor of metalloproteinase 3 (Sorsby fundus dystrophy, pseudoinflammatory) (TIMP3), mRNA	NM_000362	TIMP3	0.0009	0.715
18	Cyclin D1, a G1-S-specific cyclin, regulates G1 cyclin dependent kinase activity, involved in control of the G1-S cell cycle transition, stimulates cell proliferation, regulates apoptosis in response to DNA damage, may act as a putative proto-oncogene	I_929264	CCND1	0.0015	0.7139
19	Homo sapiens, clone IMAGE:4286250, mRNA	BC029457	BC029457	0.0005	0.6825
20	Homo sapiens hypothetical protein MGC17528 (MGC17528), mRNA	NM_080388	S100A16	0.0016	0.6589
21	Homo sapiens ribosomal protein S27-like (RPS27L), mRNA	NM_015920	RPS27L	0.0013	0.6261
22	Unknown	THC1551429	THC1551429	0.0033	0.616
23	Homo sapiens flavoprotein oxidoreductase MICAL2 (MICAL2), mRNA	NM_014632	MICAL2	0.0012	0.6143
24	Homo sapiens cDNA: FLJ22425 fis, clone HRC08686	AK026078	AK026078	0.002	0.6127
25	Homo sapiens metallothionein 2A, mRNA (cDNA clone MGC:12397 IMAGE:4051220), complete cds	BC007034	BC007034	0.0048	0.608
26	Member of the LEM3 (ligand-effect modulator 3) family or CDC50 family, has moderate similarity to S. cerevisiae Cdc50p, which generates the cell-division-cycle (CDC) phenotype following loss of function	I_957265	I_957265	0.0034	0.5992
27	Homo sapiens cold inducible RNA binding protein (CIRBP), mRNA	NM_001280	CIRBP	0.0004	0.5975
28	Unknown	THC1591470	THC1591470	0.0048	0.5838
29	Homo sapiens tumor necrosis factor receptor superfamily, member 6 (TNFRSF6), transcript variant 1, mRNA	NM_000043	TNFRSF6	0.0034	0.5819
30	Homo sapiens metallothionein 2A (MT2A), mRNA	NM_005953	MT2A	0.0022	0.5696
31	602499118F1 NIH_MGC_75 Homo sapiens cDNA clone IMAGE:4612564 5, MRNA sequence	BG427399	BG427399	0.0008	0.5622
32	Homo sapiens cDNA clone IMAGE:5504902, partial cds	BC040043	BC040043	0.0022	0.5621
33	Glioma pathogenesis-related 1, a putative secreted protein that induces apoptosis	I_964747	I_964747	0.0016	0.5609
34	Unknown	XM_165930	XM_165930	0.0012	0.5494
35	Homo sapiens immediate early response 5 (IER5), mRNA	NM_016545	IER5	0.0017	0.5481
36	Protein of unknown function	I_958029	I_958029	0.002	0.5328
37	Homo sapiens annexin A1 (ANXA1), mRNA	NM_000700	ANXA1	0.0019	0.5279
38	Homo sapiens dual specificity phosphatase 14 (DUSP14), mRNA	NM_007026	DUSP14	0.0017	0.5254
39	Homo sapiens chromosome 11 hypothetical protein ORF3 (LOC56851), mRNA	NM_020154	LOC56851	0.0017	0.5136
40	Homo sapiens calpain 2, (m/II) large subunit (CAPN2), mRNA	NM_001748	CAPN2	0.0022	0.5127
41	Homo sapiens follistatin (FST), transcript variant FST344, mRNA	NM_013409	FST	0.0037	0.5068
42	Homo sapiens cDNA FLJ11041 fis, clone PLACE1004405	AK001903	AK001903	0.0027	0.5043
43	Homo sapiens mRNA for TGF-betaIIR alpha, complete cds	D50683	D50683	0.0017	0.4985
44	Homo sapiens MEGF11 protein (MEGF11), mRNA	NM_032445	MEGF11	0.0029	0.4868
45	Homo sapiens cDNA: FLJ23015 fis, clone LNG00818	AK026668	AK026668	0.0021	0.4859
46	Homo sapiens K+ channel tetramerization protein (GMRP-1), mRNA	NM_032320	GMRP-1	0.0039	0.4742
47	Homo sapiens Shwachman-Bodian-Diamond syndrome (SBDS), mRNA	NM_016038	SBDS	0.0031	0.4709
48	Homo sapiens hypothetical protein FLJ14054 (FLJ14054), mRNA	NM_024563	FLJ14054	0.0013	0.4682
49	Homo sapiens serum/glucocorticoid regulated kinase (SGK), mRNA	NM_005627	SGK	0.0013	0.4611
50	Homo sapiens calpastatin (CAST), transcript variant 2, mRNA	NM_173060	CAST	0.0024	0.4432
51	Unknown	THC1537097	THC1537097	0.0021	0.4402
52	Homo sapiens transducer of ERBB2, 1 (TOB1), mRNA	NM_005749	TOB1	0.003	0.423
53	Homo sapiens brain protein 44-like (BRP44L), mRNA	NM_016098	BRP44L	0.0037	0.4204
54	Homo sapiens cadherin 13, H-cadherin (heart) (CDH13), mRNA	NM_001257	CDH13	0.002	0.4132
55	Homo sapiens ribosomal protein L15 (RPL15), mRNA	NM_002948	RPL15	0.002	0.401
56	Plasminogen activator inhibitor 1, a member of the serpin family of serine proteases and inhibitors, plays a role in regulating blood coagulation by inhibiting fibrinolysis, contributes to tumor progression and is a risk factor for cardiovascular	I_929648	I_929648	0.0023	0.3985
57	Homo sapiens chromosome 14 open reading frame 31 (C14orf31), mRNA	NM_152330	C14orf31	0.0047	0.3972
58	Homo sapiens coatomer protein complex, subunit zeta 2 (COPZ2), mRNA	NM_016429	COPZ2	0.0026	0.3912
59	Homo sapiens retinal short-chain dehydrogenase/reductase 4 (retSDR4), mRNA	NM_016029	DHRS7	0.0025	0.3905
60	Homo sapiens annexin A7 (ANXA7), transcript variant 2, mRNA	NM_004034	ANXA7	0.0024	0.3852
61	Homo sapiens alcohol dehydrogenase 5 (class III), chi polypeptide (ADH5), mRNA	NM_000671	ADH5	0.0042	0.3737
62	Homo sapiens v-ral simian leukemia viral oncogene homolog A (ras related), mRNA (cDNA clone MGC:48949 IMAGE:5495399), complete cds	BC039858	BC039858	0.0029	0.3726
63	Homo sapiens ras homolog gene family, member E (ARHE), mRNA	NM_005168	ARHE	0.0036	0.3722
64	Homo sapiens cDNA clone MGC:13041 IMAGE:3623612, complete cds	BC019348	BC019348	0.0032	0.3679
65	Protein containing an ADP-ribosylation factor (ARF) family domain, has low similarity to ADP-ribosylation factor 6 (human ARF6), which is involved in vesicle-mediated transport and cholera toxin activation	I_963259	I_963259	0.0033	0.3645
	RNA polymerase II K, a subunit of RNA polymerase II involved in RNA polymerase II transcription, interacts strongly				

71 Down-regulated Significant Genes (ANOVA, p-value less than 0.005)

NN	Description	GB accession	Gene symbol	Parametric p-value	Log-fold change (^{125}IUdR/^{127}IUdR)
1	603079953F1 NIH_MGC_119 Homo sapiens cDNA clone IMAGE:5171796 5, MRNA sequence	BI832240	BI832240	0.004	-0.3416
2	Unknown	ENST00000326259	ENST00000326259	0.0046	-0.3425
3	Homo sapiens hypothetical protein FLJ10156 (FLJ10156), mRNA	NM_019013	FLJ10156	0.0042	-0.3439
4	Homo sapiens lumican (LUM), mRNA	NM_002345	LUM	0.0044	-0.3498
5	Homo sapiens cDNA clone MGC:14761 IMAGE:4284687, complete cds	BC007108	NONO	0.0048	-0.3565
6	Homo sapiens chaperonin containing TCP1, subunit 4 (delta) (CCT4), mRNA	NM_006430	CCT4	0.0034	-0.3637
7	Homo sapiens ubiquitin specific protease 1 (USP1), mRNA	NM_003368	USP1	0.0032	-0.3666
8	Homo sapiens cDNA FLJ30550 fis, clone BRAWH2001502	AK055112	AK055112	0.0033	-0.3692
9	Homo sapiens SMC2 structural maintenance of chromosomes 2-like 1 (yeast) (SMC2L1), mRNA	NM_006444	SMC2L1	0.0043	-0.3696
10	Unknown	ENST00000329543	ENST00000329543	0.0044	-0.3729
11	Unknown	ENST00000333189	ENST00000333189	0.0037	-0.3813
12	Homo sapiens baculoviral IAP repeat-containing 5 (survivin), mRNA (cDNA clone IMAGE:3506845), partial cds	BC000784	BC000784	0.0039	-0.3826
13	Homo sapiens similar to peptidylprolyl isomerase A (cyclophilin A) [Homo sapiens] (COAS2), mRNA	NM_178230	COAS2	0.0037	-0.3833
14	Homo sapiens cDNA FLJ36593 fis, clone TRACH2014077, highly similar to Homo sapiens mRNA for NS1-binding protein	AK093912	IVNS1ABP	0.0038	-0.3851
15	Homo sapiens mRNA; cDNA DKFZp434I225 (from clone DKFZp434I225); partial cds	AL117637	AL117637	0.0029	-0.3861
16	Homo sapiens histone 1, H2ag (HIST1H2AG), mRNA	NM_021064	HIST1H2AG	0.0045	-0.3906
17	Homo sapiens kinesin family member 23 (KIF23), transcript variant 1, mRNA	NM_138555	KIF23	0.0045	-0.392
18	Homo sapiens collagen, type XVI, alpha 1 (COL16A1), mRNA	NM_001856	COL16A1	0.0034	-0.399
19	Unknown	ENST00000333626	ENST00000333626	0.0037	-0.4011
20	Unknown	ENST00000329485	ENST00000329485	0.0038	-0.4037
21	Homo sapiens histone 1, H2bi (HIST1H2BI), mRNA	NM_003525	HIST1H2BI	0.0039	-0.4088
22	Homo sapiens histone 1, H2bd (HIST1H2BD), transcript variant 1, mRNA	NM_021063	HIST1H2BD	0.0019	-0.4127
23	Homo sapiens kinesin family member 2C (KIF2C), mRNA	NM_006845	KIF2C	0.0017	-0.4197
24	Unknown	ENST00000333472	ENST00000333472	0.0015	-0.4296
25	Unknown	THC1595926	THC1595926	0.0043	-0.4323
26	Unknown	THC1516237	THC1516237	0.0044	-0.4323
27	Homo sapiens heterogeneous nuclear ribonucleoprotein F (HNRPF), mRNA	NM_004966	HNRPF	0.0022	-0.4341
28	Homo sapiens eukaryotic translation initiation factor 3, subunit 6 48kDa (EIF3S6), mRNA	NM_001568	EIF3S6	0.0019	-0.4382
29	Homo sapiens histone 1, H2bj (HIST1H2BJ), mRNA	NM_021058	HIST1H2BJ	0.0046	-0.4488
30	Human translation initiation factor 5 (eIF5) mRNA, complete cds	U49436	U49436	0.0021	-0.4568
31	Homo sapiens histone 2, H4 (HIST2H4), mRNA	NM_003548	HIST2H4	0.0038	-0.4634
32	Homo sapiens Ras-GTPase-activating protein SH3-domain-binding protein (G3BP), mRNA	NM_005754	G3BP	0.004	-0.4716
33	Homo sapiens c-myc binding protein (MBP-1) mRNA, complete cds	M55914	ENO1	0.0013	-0.4724
34	Unknown	ENST00000327414	ENST00000327414	0.0015	-0.477
35	Homo sapiens histone 1, H2bo (HIST1H2BO), mRNA	NM_003527	HIST1H2BO	0.0012	-0.4798
36	Homo sapiens Wolf-Hirschhorn syndrome candidate 1 (WHSC1), transcript variant 4, mRNA	NM_014919	WHSC1	0.0043	-0.48
37	Homo sapiens mRNA full length insert cDNA clone EUROIMAGE 1913076	AL359062	AL359062	0.0033	-0.484
38	Homo sapiens actin, alpha, cardiac muscle (ACTC), mRNA	NM_005159	ACTC	0.0022	-0.4865
39	Homo sapiens spermine synthase (SMS), mRNA	NM_004595	SMS	0.0019	-0.488
40	Homo sapiens arylsulfatase B, transcript variant 2, mRNA (cDNA clone MGC:34518 IMAGE:5186657), complete cds	BC029051	BC029051	0.002	-0.4904
41	Homo sapiens histone 2, H2be, mRNA (cDNA clone IMAGE:2989788)	BC005827	BC005827	0.0011	-0.4979
42	Homo sapiens clone TCCCTA00084 mRNA sequence	AY007110	AY007110	0.0012	-0.5082
43	RFX gene	A20498	A20498	0.0048	-0.5093
44	Homo sapiens heat shock 70kDa protein 2 (HSPA2), mRNA	NM_021979	HSPA2	0.0029	-0.5192
45	Homo sapiens cyclin A2 (CCNA2), mRNA	NM_001237	CCNA2	0.0026	-0.5237
46	Homo sapiens histone 1, H4h (HIST1H4H), mRNA	NM_003543	HIST1H4H	0.0016	-0.524
47	Homo sapiens polo-like kinase (Drosophila) (PLK), mRNA	NM_005030	PLK	0.0044	-0.528
48	Homo sapiens kinesin family member 20A (KIF20A), mRNA	NM_005733	KIF20A	0.0032	-0.5313
49	Unknown	ENST00000330382	ENST00000330382	0.0016	-0.5326
50	Unknown	ENST00000294851	ENST00000294851	0.0019	-0.5401
51	Homo sapiens, Similar to heparan sulfate 6-O-sulfotransferase, clone IMAGE:3355592, mRNA, partial cds	BC001196	BC001196	0.0045	-0.5412
52	Human asparagine synthetase mRNA, complete cds	M27396	M27396	0.0023	-0.5427
53	Unknown	ENST00000329768	ENST00000329768	0.003	-0.5474
54	AUTO: Protein of unknown function	I_1961525	I_1961525	0.0009	-0.5534
55	Homo sapiens mRNA; cDNA DKFZp434M0223 (from clone DKFZp434M0223); partial cds	AL137479	AL137479	0.0015	-0.5724
56	Homo sapiens CES hBr3 mRNA for brain carboxylesterase hBr3, complete cds	AB025028	AB025028	0.0049	-0.5885
57	Homo sapiens chromosome 10 open reading frame 3 (C10orf3), mRNA	NM_018131	C10orf3	0.0007	-0.5921
58	Homo sapiens clone FLC1492 PRO3121 mRNA, complete cds	AF130082	COL3A1	0.0021	-0.595
59	Homo sapiens tumor rejection antigen (gp96) 1, mRNA (cDNA clone IMAGE:3938823), complete cds	BC009195	BC009195	0.0019	-0.6018
60	Homo sapiens histone 1, H2ad (HIST1H2AD), mRNA	NM_021065	HIST1H2AD	0.0027	-0.6189
61	Homo sapiens nicotinamide N-methyltransferase (NNMT), mRNA	NM_006169	NNMT	0.0008	-0.6321
62	Homo sapiens platelet-derived growth factor receptor, beta polypeptide (PDGFRB), mRNA	NM_002609	PDGFRB	0.002	-0.6504
63	Homo sapiens dihydropyrimidinase-like 2 (DPYSL2), mRNA	NM_001386	DPYSL2	0.0005	-0.6505
64	Homo sapiens histone 1, H2aj (HIST1H2AJ), mRNA	NM_021066	HIST1H2AJ	0.0037	-0.6546
65	Homo sapiens histone 1, H4b (HIST1H4B), mRNA	NM_003544	HIST1H4B	0.0003	-0.6637
66	Human mRNA for pro alpha 1 (III) collagen C-terminal propeptide	X01742	COL3A1	0.0015	-0.6908
67	Homo sapiens cDNA FLJ37575 fis, clone BRCOC2003125, moderately similar to TRIOSEPHOSPHATE ISOMERASE (EC 5.3.1.1)	AK094894	AK094894	0.0023	-0.6938
68	[illegible]	[illegible]	[illegible]	[illegible]	[illegible]
